# Aromatic Bis[aminomethylidenebis(phosphonic)] Acids Prevent Ovariectomy-Induced Bone Loss and Suppress Osteoclastogenesis in Mice

**DOI:** 10.3390/ijms22179590

**Published:** 2021-09-03

**Authors:** Anna Nasulewicz-Goldeman, Waldemar Goldeman, Anna Nikodem, Marcin Nowak, Diana Papiernik, Tomasz M. Goszczyński, Joanna Wietrzyk

**Affiliations:** 1 Department of Experimental Oncology, Hirszfeld Institute of Immunology and Experimental Therapy, Polish Academy of Sciences, 53-114 Wroclaw, Poland; diana.papiernik@gmail.com (D.P.); goszczynski@hirszfeld.pl (T.M.G.); joanna.wietrzyk@hirszfeld.pl (J.W.); 2 Department of Organic and Medicinal Chemistry, Wrocław University of Science and Technology, Wybrzeze Wyspianskiego 27, 50-370 Wrocław, Poland; waldemar.goldeman@pwr.edu.pl; 3 Department of Mechanics, Materials and Biomedical Engneering, Faculty of Mechanical Engineering, Wrocław University of Science and Technology, Smoluchowskiego 25, 50-370 Wrocław, Poland; anna.nikodem@pwr.edu.pl; 4 Faculty of Veterinary Medicine, Wroclaw University of Environmental and Life Sciences, Norwida 31, 50-366 Wroclaw, Poland; marcin.nowak@upwr.edu.pl

**Keywords:** osteoporosis, aminobisphosphonates, osteoclastogenesis, antiresorptive activity, ovariectomy

## Abstract

Osteoporosis is a skeletal disease associated with excessive bone turnover. Among the compounds with antiresorptive activity, nitrogen-containing bisphosphonates play the most important role in antiosteoporotic treatment. In previous studies, we obtained two aminomethylidenebisphosphonates—benzene-1,4-bis[aminomethylidene(bisphosphonic)] (WG12399C) acid and naphthalene-1,5-bis[aminomethylidene(bisphosphonic)] (WG12592A) acid—which showed a significant antiproliferative activity toward J774E macrophages, a model of osteoclast precursors. The aim of these studies was to evaluate the antiresorptive activity of these aminobisphosphonates in ovariectomized (OVX) Balb/c mice. The influence of WG12399C and WG12592A administration on bone microstructure and bone strength was studied. Intravenous injections of WG12399C and WG12592A bisphosphonates remarkably prevented OVX-induced bone loss; for example, they sustained bone mineral density at control levels and restored other bone parameters such as trabecular separation. This was accompanied by a remarkable reduction in the number of TRAP-positive cells in bone tissue. However, a significant improvement in the quality of bone structure did not correlate with a parallel increase in bone strength. In ex vivo studies, WG12399C and WG12592A remarkably bisphosphonates reduced osteoclastogenesis and partially inhibited the resorptive activity of mature osteoclasts. Our results show interesting biological activity of two aminobisphosphonates, which may be of interest in the context of antiresorptive therapy.

## 1. Introduction

Osteoporosis is a skeletal disorder characterized by the imbalance between bone formation and resorption, which leads to a decrease in bone mass and density and to progressive changes in bone structure [1]. This, in turn, results in a higher risk of bone fracture, most commonly in the vertebrae, wrist, or hip [2]. Currently, osteoporosis is a global health problem affecting an estimated 200 million people [3]. Aging and chronic estrogen deficiency are the most important risk factors for osteoporosis [4,5]. Numerous therapeutic agents have been developed to treat osteoporosis. These include either bone-anabolic or antiresorptive drugs [6]. Among these drugs, the most active compounds belong to the class of nitrogen-containing bisphosphonates (N-BPs). Because of their chemical structure, BPs efficiently accumulate in the bone tissue and reduce bone resorption through the inhibition of osteoclast activity [7]. In most commercial BPs, a hydroxyl group that is covalently bound to the carbon atom of the bisphosphonic group (P-C-P) as the R^1^ substituent determines the high affinity of BPs to bone mineral surface, while the antiresorptive activity is dependent basically on the chemical structure of the R^2^ chain [8,9]. The acidic microenvironment generated by resorbing osteoclasts facilitates a release of BPs from bone tissue in osteoclast lacunae, which allows their internalization into osteoclasts by endocytosis [10,11]. The molecular mechanism of action of N-BPs involves the prevention of farnesyl pyrophosphate (FPP) and geranylgeranyl pyrophosphate (GGPP) through the inhibition of the mevalonate pathway [12].

**Figure 1:**
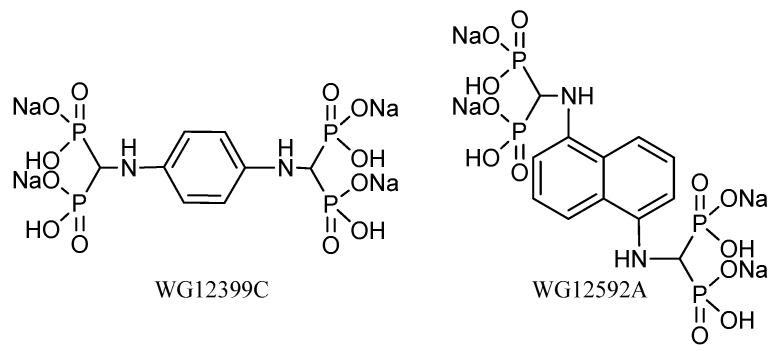
Chemical structures of the tetrasodium salts of benzene-1,4-bis[aminomethylidene(bisphosphonic)] acid and naphthalene-1,5-bis[aminomethylidene(bisphosphonic)] acid.

In our previous studies, we obtained two new compounds belonging to the group of aminomethylidenebisphosphonates: Benzene-1,4-bis[aminomethylidene(bisphosphonic)] acid and naphthalene-1,5-bis[aminomethylidene(bisphosphonic)] acid [13]. These BPs have two bisphosphonic groups in their structures and aromatic ring (phenyl and naphthyl, respectively) bonded directly to the nitrogen atom of the aminomethylidenebisphosphonic group. Both BPs showed a significant antiproliferative activity toward murine J774E macrophages, a model of osteoclast precursors in vitro, through changes in cell cycle progression. Moreover, these compounds enhanced the cytotoxic activity of doxorubicin and cisplatin, especially when applied prior to cytostatics, and showed proapoptotic activity in osteoclast precursors, which was manifested by an increase in caspase-3 activity and percentage of apoptotic cells [14]. The results obtained prompted us to perform an extended research study on the biological activity of these BPs. Because of the low solubility of the free acids, both compounds were used in the present study as their tetrasodium salt (Figure 1).

The specific aim of this study was to evaluate the in vivo antiresorptive activity of WG12399C and WG12592A aminobisphosphonates. Ovariectomized (OVX) Balb/c mice were used in this study as a model of estrogen deficiency-related osteoporosis. Female OVX mice and rats that mimic the postmenopausal hormonal status of women are the most widely used animal model of osteoporosis for antiosteoporotic drug screening [15,16]. The influence of WG12399C and WG12592A administration on bone microstructure, bone strength, and biochemical parameters of bone turnover was studied. The ex vivo antiresorptive activity of aminobisphosphonates in osteoclasts was also evaluated.

## 2. Results

### 2.1. In Vivo Toxicity of WG12399C and WG12592A

The mortality rate as well as the overall acute toxicity of intravenously administered bisphosphonates increased progressively starting from the dose 55 mg/kg for WG12399C and 3.5 mg/kg for WG12592A. LD_50_ doses estimated based on the dose-response curves were 73.8 mg/kg for WG12399C and 3.3 mg/kg for WG12592A. On the basis of these doses, the concentrations of compounds for the subacute toxicity studies were determined. WG12399C and WG12592A were administered intravenously with the following total doses divided into four weekly injections: WG12399C—18, 35 and 50 mg/kg; WG12592A—1, 2 and 4 mg/kg. No clinical signs of toxicity were observed in any of the groups tested. All of the mice showed normal behavior and vitality, and no deaths were recorded. The body weight of the animals in each group increased with time and the weight gain in the course of the experiment did not differ significantly among the groups (Figure S7). Finally, the gross necropsy did not show any macroscopic changes in the morphology of the internal organs. Based on these results the following weekly doses proved to be well tolerated in animals: 12.5 mg/kg for WG12399C and 1 mg/kg for WG12592A.

### 2.2. Aminobisphosphonates Prevent OVX-Induced Bone Loss

**Figure 2:**
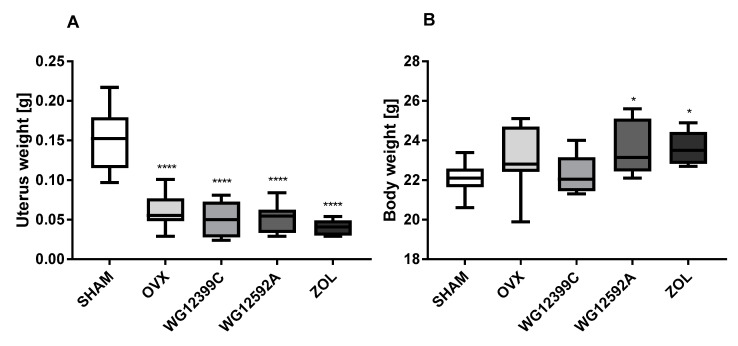
Changes in the uterus (**A**) and body weight (**B**) in ovariectomized mice. Mice were bilaterally ovariectomized (OVX) and then administered intravenously with WG12399C 60 mg/kg, WG12592A 6 mg/kg, or zoledronate 120 μg/kg divided into six weekly doses. Vehicle-treated (OVX) and sham-operated (SHAM) mice served as controls. After the final dose, mice were sacrificed, and their uteruses were isolated and weighted immediately. In the box plots, boxes represent the 75th to 25th percentiles, the horizontal line in the box is the median, and the whiskers the maximum and minimum values, *n* = 8; * *p* < 0.05 and **** *p* < 0.0001 vs. SHAM group were assessed with one-way analysis of variance.

It has been suggested that postmenopausal osteoporosis is closely related to estrogen deficiency, accompanied with increased osteoclast formation and enhanced bone resorption [17]. In our studies, bilateral ovariectomy caused a significant depletion of estrogen in mice, which resulted in a significant involution of the uterus (Figure 2A). The mean uterus weight in groups of OVX mice was decreased by 60% to 73% as compared to their age-matched sham controls. The baseline body weight at the beginning of the experiment did not differ significantly among groups. In the course of the study the average body weight increased by 7 to 14% (Figure 2B). The differences were not statistically significant in the OVX and WG12399C groups; however, it reached a significant level in zoledronate- and WG12592A-treated mice as compared to SHAM animals.

**Figure 3:**
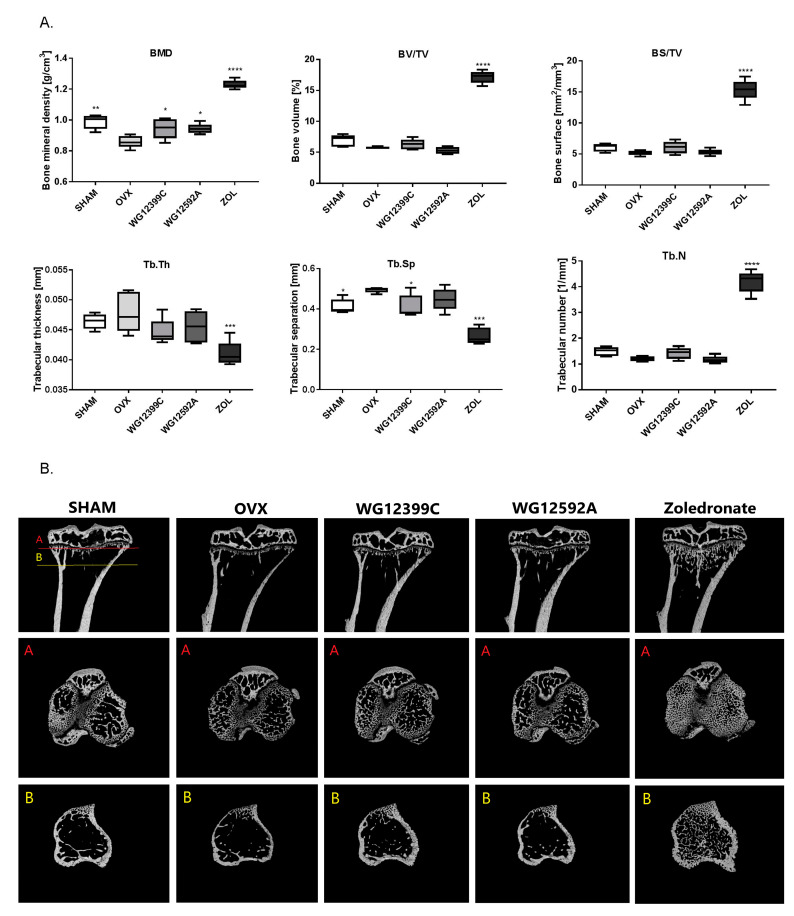
The effect of bisphosphonates on trabecular bone architecture of the proximal tibial metaphysis. Mice were bilaterally ovariectomized (OVX) and then administered intravenously with WG12399C 60 mg/kg, WG12592A 6 mg/kg, or zoledronate 120 μg/kg divided into six weekly doses. Vehicle-treated (OVX) and sham-operated (SHAM) mice served as controls. (**A**) Histograms representing the structural parameters of the trabecular bone measured with µCT: BMD—bone mineral density; BV/TV—bone volume, BS/TV—bone surface, Tb.Th—trabecular thickness, Tb.Sp—trabecular separation, Tb.N—trabecular number. In the box plots, boxes represent the 75th to 25th percentiles, the horizontal line in the box is the median, and the whiskers the maximum and minimum values, *n* = 5; * *p* < 0.05, ** *p* < 0.01, *** *p* < 0.001, and **** *p* < 0.0001 vs. the OVX group were assessed with one-way analysis of variance. (**B**) µ-CT images of the proximal tibias (upper row, longitudinal view; two bottom rows, axial view of the metaphyseal region).

On day 56 after ovariectomy, mice were sacrificed, and their tibias were excised for μCT analysis. The results indicated that OVX caused a significant loss of trabecular bone in mice eight weeks after surgery. The analysis of trabecular bone in the proximal tibia demonstrated that BMD decreased, whereas Tb.Sp increased significantly in OVX mice as compared to those in the sham-operated group (Figure 3A). Furthermore, BV/TV, BS/TV, and Tb.N values were reduced; however, the difference was not statistically significant. Figure 3B shows the representative µCT images of tibial sections from the experimental groups. Treatment with reference zoledronate at the dose of 120 μg/kg provided a complete protection against OVX-induced bone loss. Zoledronate caused a remarkable increase in BMD, BV/TV, BS/TV, and Tb.N. In contrast, Tb.Sp and Tb.Th were significantly reduced as compared to those in the OVX and SHAM groups. This may indicate that newly formed bone trabeculae are thinner then control ones; however, this is compensated by a significant increase in the number of trabeculae.

The effects of treatment with WG12399C at the dose of 60 µg/kg were not that profound; however, the overall cancellous bone microarchitecture remained at the level of that found in sham-operated healthy mice. Moreover, a significant increase in cancellous BMD and a significant decrease in Tb.Sp values were observed as compared to those in OVX mice. The administration of WG12592A BP improved BMD; however, changes in the histomorphometric parameters of trabecular bone were not significant.

### 2.3. The Influence of Aminobisphosphonates on Bone Biomechanics

The results of mechanical tests performed using the four-point bending test showed a decrease in the mean values of most mechanical parameters of the bone tissue in mice treated with WG12399C as compared to those in the OVX group. For WG12592A compound, ultimate strength (σ_ult_) decreased and the modulus of elasticity (E value) increased (Table 1). No significant changes in the mean value of the stiffness (k) were observed in the treated groups. The highest values of the area moment of inertia were recorded for bones from animals treated with WG12592A and reference zoledronate (Table 2).

### 2.4. Histological Analysis of Bone

**Figure 4:**
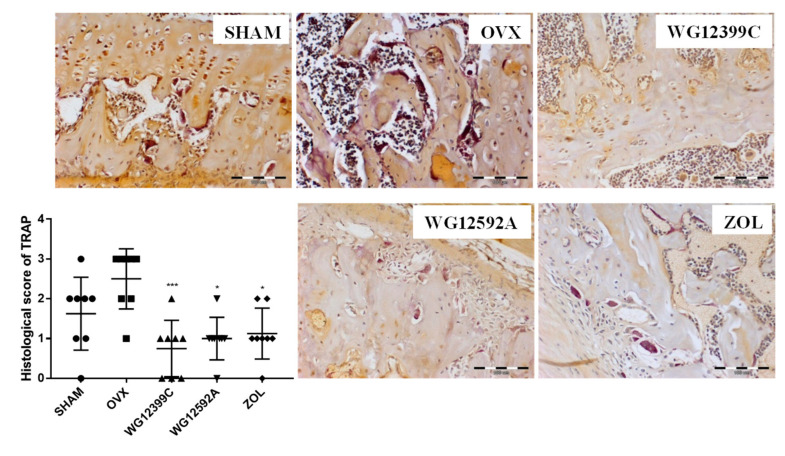
The effect of bisphosphonates on bone resorption. Pictures represent sections of TRAP-stained metaphyseal regions of the proximal tibias. Scale bars = 100 µm. Histogram represents histological score for TRAP staining calculated with CellA software. Data are expressed in scatter plots, the horizontal line in the plots is the mean, and the whiskers are SD values, *n* = 8; * *p* < 0.05 and *** *p* < 0.001 vs. the OVX group were assessed with Kruskal–Wallis test.

The results obtained posed the question of whether WG12399C and WG12952A prevent ovariectomy-induced bone loss by inhibiting osteoclast differentiation. To address this issue, we stained histological sections of proximal tibial metaphysis for TRAP activity, which is a marker of resorbing osteoclasts. Histological scoring of TRAP staining showed an increased number of TRAP-positive osteoclasts in the OVX group as compared to that in the SHAM group. However, treatment with BPs significantly reduced the number of large multinucleated TRAP-positive cells when compared with that in the OVX group. These results are summarized in Figure 4.

### 2.5. Plasma Calcium and Bone Turnover Markers

**Figure 5:**
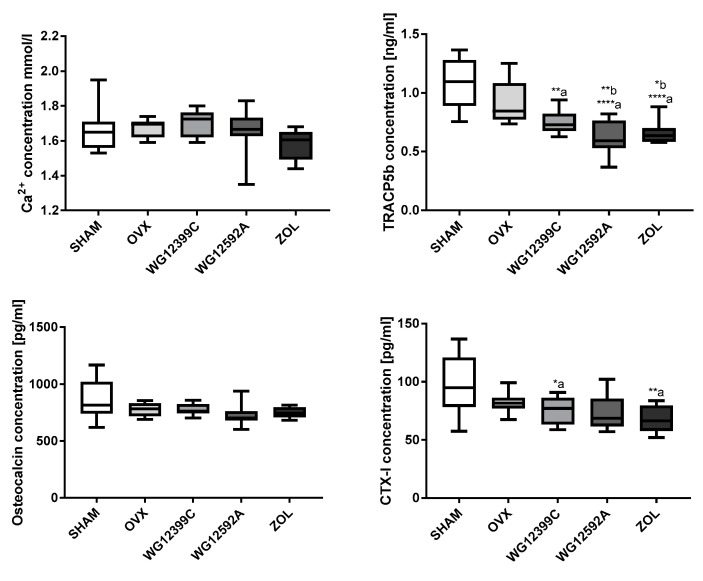
The level of Ca^2+^ and bone turnover markers in plasma of mice administered with bisphosphonates. Mice were bilaterally ovariectomized (OVX) and then treated with WG12399C 60 mg/kg, WG12592A 6 mg/kg, or zoledronate 120 μg/kg divided into six weekly doses. Vehicle-treated (OVX) and sham-operated (SHAM) mice served as controls. Ca^2+^ level was measured in plasma by using the Cobas c 111 device. TRACP5b, OC, and CTX-I levels were estimated in plasma by using the CLIA kits. In the box plots, boxes represent the 75th to 25th percentiles, the horizontal line in the box is the median, and the whiskers the maximum and minimum values, *n* = 8; * *p* < 0.05, ** *p* < 0.01, **** *p* < 0.0001, a—significant vs. the SHAM group, b—significant vs. the OVX group, assessed with one-way analysis of variance.

The plasma levels of Ca^2+^, OC, TRACP5b, and CTX-I as bone turnover markers are summarized in Figure 5. Plasma calcium and OC levels did not differ significantly between controls and mice treated with WG12399C, WG12592A, or zoledronate. In contrast, a remarkable reduction in plasma TRACP5b activity was observed in all the groups of mice treated with BPs. Moreover, plasma levels of CTX-I, another bone resorption marker, were significantly decreased in mice treated with WG12399C or zoledronate as compared to those in both OVX and sham-operated groups.

### 2.6. Osteoclast Differentiation from OC Precursors

**Figure 6:**
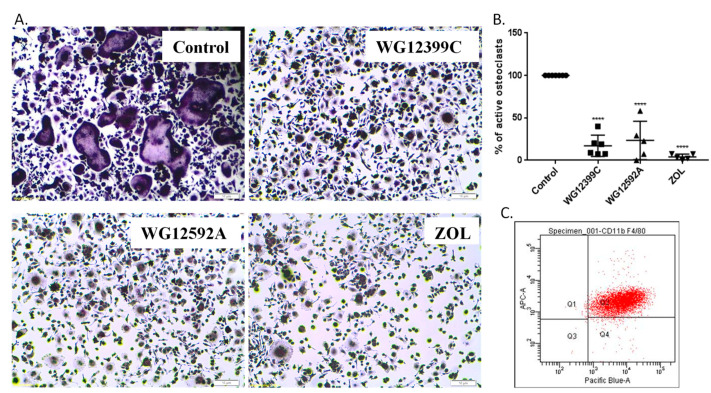
WG12399C and WG12592A bisphosphonates inhibit osteoclast maturation. BMDMs were treated with 0.919 μM WG12399C, 0.421 µM WG12592A, or 0.345 μM zoledronate for eight days. TRAP-positive cells with at least three nuclei were counted as active osteoclasts. (**A**) Representative images of TRAP-stained osteoclasts. (**B**) Histogram representing the percentage of active osteoclasts compared to control group. Data are expressed in scatter plots, the horizontal line in the plots is the mean, and the whiskers are SD values; **** *p* < 0.0001 vs. the control group were assessed with one-way analysis of variance. (**C**) Representative dot plot showing the purity of BMDMs. Cells were fluorescently labeled with CD11b and F4/80 antibodies. The experiment was performed five times.

We also examined the effect of BPs on osteoclastogenesis in vitro. Primary bone marrow cells were differentiated for four days and tested for purity. Over 98% of cells expressed both CD11b and F4/80 markers (Figure 6C). Fully differentiated macrophages were then cultured in the presence of RANKL and M-CSF and treated with BPs. TRAP staining revealed that both WG12399C and WG12592A BPs remarkably inhibited osteoclastogenesis. The percentage of activated osteoclasts was reduced by 83% for WG12399C and by 77% for WG12592A compound as compared to that in untreated control cells (Figure 6B).

### 2.7. Resorptive Activity of Osteoclasts In Vitro

**Figure 7:**
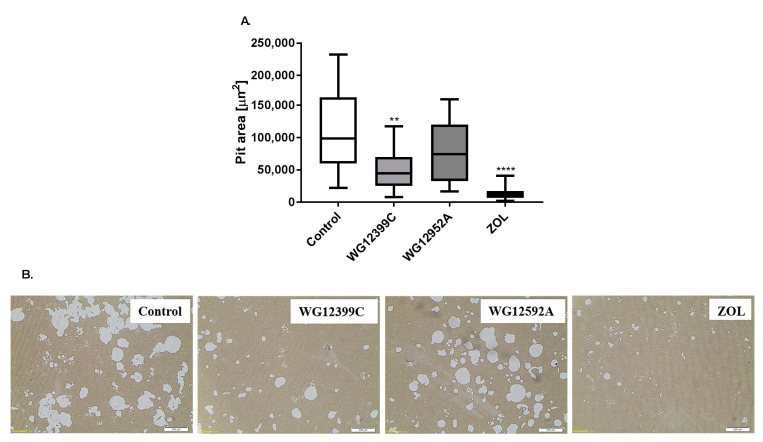
WG12399C and WG12592A bisphosphonates inhibit resorptive activity of osteoclasts. BMDMs were treated with 1.84 μM WG12399C, 0.842 µM WG12592A, or 0.345 μM zoledronate for 12 days. (**A**) Histogram representing the percentage of active osteoclasts compared to that in the control group. In the box plots, boxes represent the 75th to 25th percentiles, the horizontal line in the box is the median, and the whiskers the maximum and minimum values; scales bars = 200 µm, ** *p* < 0.01 and **** *p* < 0.0001 vs. the OVX group were assessed with Kruskal–Wallis test. (**B**) Representative images of TRAP-stained osteoclasts. The experiment was performed five times.

Together with the inhibitory effect on the differentiation of osteoclasts, WG12399C and WG12592A were tested for their antiresorptive activity. Quantification of the total resorption pit areas on Corning Osteo Assay Microplates showed significant differences in resorption activity between BP-treated and control untreated osteoclasts. Treatment with 1.84 μM WG12399C compound resulted in over 40% reduction in the total pit area as compared to control values. WG12592A at the concentration of 0.842 µM inhibited the resorption activity of osteoclasts by 30%, however without statistical significance (Figure 7).

## 3. Discussion

Bisphosphonates are the potent inhibitors of osteoclast-mediated bone resorption and have been widely used as effective therapeutic agents in bone disorders. Most of the clinically applied BPs are hydroxybisphosphonates. In these compounds the carbon atom of the methylenebisphosphonic group is attached directly to the hydroxyl group [18]. In current study, we evaluated the skeletal effects of two BPs which contain the methylenebisphosphonic group bounded directly to the nitrogen atom.

Because osteoporosis occurs most frequently in postmenopausal women due to severe estrogen deficiency, an ovariectomized (OVX) mouse model was used in the study. Our results confirmed that bilateral ovariectomy causes a significant depletion of estrogen manifested by uterus involution in all OVX mice. In addition, significant changes in trabecular bone microarchitecture were also observed in mice subjected to OVX. In the analysis of 3D structure of proximal tibial metaphysis, a decrease in BMD and an increase in Tb.Sp values induced by OVX were evident. Unfortunately, these changes were not reflected in the plasma levels of TRAC5B and CTX-I. Based on the results obtained in this study we cannot explain this phenomenum. We postulate that this may be related to the ongoing processes of bone remodeling or simply to aging of animals, since both factors may influence the serum level of bone markers [19,20].

Many studies have shown that nitrogen-containing BPs may inhibit bone loss and preserve bone volume in both animal models and humans; however, their individual effect on bone resorption is variable [21,22,23]. Here we report that all BPs applied in the study suppressed OVX-induced bone loss. The µCT analysis revealed an improvement of important structural indices in BP-treated mice as compared to those in the untreated control group (Figure 3 and Figure S8). Intravenous injections with 60 mg/kg WG12399C or 6 mg/kg WG12592A almost completely prevented BMD decrease, and these indices remained at the level comparable to that in the sham group. Trabecular number and trabecular separation were also restored in mice receiving WG12399C and, to a lower degree, in the WG12592A group. A parallel analysis showed that treatment with WG12399C, WG12592A, or zoledronate also significantly improved the quality of bone structure in distal femoral metaphysis (Figure S8). In this experiment single doses of WG12399C and WG12592A were applied, which is the main limitation of the study. However, since zoledronate chosen as the reference compound is the most active bisphosphonate applied in the clinic, we decided to use the maximum well-tolerated doses of WG12399C and WG12592A estimated in toxicity studies and evaluate their biological activity in comparison to zoledronate.

Interestingly, a remarkable improvement of microstructure was observed in zoledronate-treated mice as compared to that in healthy sham-operated animals. This phenomenon has been previously reported in other studies. In tumor-bearing mice, zoledronate caused a significant increase in bone volume in both OVX and sham-operated animals compared to that in the respective controls; this demonstrated that bone resorption is reduced to the same extent in the models of pre- and postmenopausal bone [24]. Similarly, Kim et al. showed that treatment with zoledronate spectacularly improved BMD as well as bone volume and thickness in a murine model of irradiation-induced bone loss [25]. It is also worth noting that although the overall bone microstructure in zoledronate-treated mice was improved as compared to that in the other experimental groups, the trabecular thickness was reduced in both tibial (Figure 3A) and femoral metaphyses (Figure S8). This was compensated by a significant increase in the number of probably newly formed bone trabeculae, which resulted in a decrease in Tb.Sp values.

Bone mass is a major determinant of bone strength, and BMD is considered to be the standard parameter to evaluate bone strength and the risk of bone fractures. However, other factors related to bone microstructure also contribute to bone integrity and its resistance to mechanical stress. These factors include trabecular thickness and separation, porosity, and collagen content [26]. Thicker plate-like trabeculae provide better bone quality and strength than thinner rod-like trabeculae [27]. Thus, the bone quality defined as the sum of structural and material properties rather than BMD alone seems to be a better predictor of bone strength.

In these studies, mechanical tests were performed using the four-point bending test, the main advantage of which is the constant bending moment in the area of the examined bone (middle region) [28]. Unexpectedly, a significant improvement in the quality of bone microstructure in tibial and femoral metaphysis did not correlate with an improvement of parallel indices in femoral shaft (Figure S9) and with an increase in bone strength in our study. This may be related to the fact the long bone is very diversified along its entire length, both in terms of shape and size of the cross-section, which is determined precisely by the thickness of the cortex layer and the cross-section diameter. A bone fracture always occurs in the weakest place, often below the distal femoral epiphysis, where a cortical layer of fairly large diameter but a small thickness is observed. Moreover, because of their chemical structure, BPs show high affinity to bone tissue and binds readily to resorbing surfaces. Therefore, they accumulate much more profusely in trabecular bone than in cortical bone [29]. This may be one of the reasons why a significant improvement in trabecular structure in the area of tibial and femoral metaphyses is not correlated with a remarkable increase of parallel parameters in bone shafts (Figure S9) as well as in bone strength in the bending test.

Bone sustains its homeostatic state by maintaining the balance between two opposite processes: Bone formation by osteoblasts and bone resorption mediated by osteoclasts. Osteoporosis is caused by an excessive osteoclast-related osteolysis. Estrogen deficiency affects bone remodeling through direct and indirect mechanisms [30]. These mechanisms include the influence on osteoclast lifespan and apoptosis [31], RANKL-induced osteoclast differentiation [32], and the effects on the production of RANKL [33] and osteoprotegrin [34]. Previous studies have shown that staining for TRAP activity reflects the increase in the activity and size of osteoclasts in OVX mice [35,36], and the inhibition of osteoclast activity is correlated with the improvement in bone microarchitecture [37,38]. Thus far, the influence of BPs on the morphology and activity of osteoclasts has been shown in many animal model studies and in bone biopsies from BPs-treated patients [39,40]. However, the exact nature of the effect of BPs on osteoclast number, activity, and life-span remains inconclusive. In our present study, the staining for TRAP activity showed an elevated number of active osteoclasts in distal tibial metaphysis in OVX mice as compared to that in sham-operated animals. Treatment with WG12399C or WG12592A significantly reduced the number of large multinucleated TRAP-positive cells when compared with that in OVX mice. These changes were at comparable level to those observed for the reference zoledronic acid.

The reduction in the TRAP activity reflects the decreased number of and suppressed resorptive activity of osteoclasts and correlates with the overall condition of bone structure. However, the exact mechanism involved in this phenomenon remains elusive.

Thus far, it has been shown that zoledronic acid decreased the number of TRAP-positive multinuclear cells derived from mouse osteoclast precursors through the inhibition of differentiation and multinucleation [41]. In turn, Hirayama et al. postulated that in rheumatoid arthritis, an increased osteoclast functional activity rather than osteoclast formation is more likely to play a role in generalized bone loss [42]. Thus, we posed the question of whether the changes in osteoclast number and activity in BP-treated mice result from the diminished maturation of osteoclast precursors or from their reduced resorptive activity.

In previous studies, we have shown that WG12399C and WG12592A significantly restrain the proliferation rate of J774E cells, which are a convenient model of osteoclast precursors for in vitro studies [13]. The antiproliferative effect of new BPs was related to the changes in cell cycle progression and proapoptotic activity [14]. However, these model cells do not readily differentiate into mature osteoclasts under standard culturing conditions. Thus, primary bone marrow cells were isolated from murine marrow and differentiated to macrophages. Then, BMDMs were cultured in the presence of RANKL and M-CSF and either WG12399C, WG12592A, or reference zoledronate. Both the studied BPs remarkably inhibited osteoclastogenesis, resulting in the reduction in the number of active, TRAP-positive osteoclasts by 83% and 77%, respectively, for WG12399C and WG12592A. The influence of these BPs on osteoclast differentiation was similar to the effects observed for the reference zoledronic acid. Then, WG12399C and WG12592A compounds were tested for their antiresorptive properties. Fully differentiated macrophages cultured on osteo-microplates in the presence of RANKL and M-CSF develop into active osteoclasts and efficiently resorb the mineral coating of the plates. In these studies, the treatment of cells with WG12399C or WG12592A resulted in partial inhibition of their resorptive activity; however, these effects were not as pronounced as for the reference zoledronate. Nevertheless, both aminomethylidenebisphosphonates significantly affected the maturation and antiresorptive activity of osteoclasts.

## 4. Materials and Methods

### 4.1. Compounds

Tetrasodium salts of benzene-1,4-bis[aminomethylidene(bisphosphonic)] acid (WG12399C) and naphthalene-1,5-bis[aminomethylidene(bisphosphonic)] acid (WG12592A) were prepared from free acids using the procedure described below. The preparation of the starting free bisphosphonic acids was carried out in a large scale using the procedure developed in our previous studies [13] and was described in detail in the Supplementary Materials. ^1^H and ^31^P NMR spectra were recorded on a 600 MHz Bruker Avance spectrometer in D_2_O as the solvent (at 600 MHz and 243 MHz, respectively) and locked on deuterium from a solvent. Chemical shifts (δ) are expressed in parts per million (ppm). High-resolution mass spectra were recorded on a Bruker MicrOTOF-QII spectrometer (Bruker Daltonic, Bremen, Germany) equipped with an electrospray ion source. The instrument was operated in the negative-ion mode and calibrated externally with a sodium formate (10 mM). The capillary temperature was 200 °C, and N_2_ was used as a nebulizing gas. Data were acquired with micrOTOFcontrol 4.0 and processed for calibration with DataAnalysis software from Daltonic GmbH (Germany). Samples of the studied compounds were infused into the mass spectrometer at a flow rate of 3 μL/min in water/isopropanol mixture (50/50, *v*/*v*). All reagents and solvents used in the synthesis were of commercial quality and purchased from Sigma-Aldrich (Darmstadt, Germany) and a local supplier (Avantor, Poland). Zoledronate was synthesized according to literature procedure [43].

Procedure for the preparation of WG12399C and WG12592A: First, 10 mmol of free benzene-1,4-bis[aminomethylidene(bisphosphonic)] acid and naphthalene-1,5-bis-[aminomethylidene(bisphosphonic)] acid were dissolved in 40 mL of 1.0 M NaOH (40 mmol). The resulting solutions were added dropwise to the vigorously stirred 96% ethanol (250 mL), and the resulting suspensions aged over night at room temperature. The suspensions were centrifuged at 4700 rpm for 30 min. After centrifugation, the obtained solid was washed with 96% ethanol (4∙25 mL) and dried in vacuo, yielding the tetrasodium salts of benzene-1,4-bis[aminomethylidene(bisphosphonic)] acid (WG12399C) and naphthalene-1,5-bis[aminomethylidene(bisphosphonic)] acid (WG12592A) with 90% and 98% yield, respectively. The structures of the obtained BPs were confirmed by ^1^H, ^31^P NMR and HRMS-ESI spectroscopy: WG12399C: ^1^H NMR (D_2_O, 600 MHz): δ 3.61 (t, 2H, *J* = 19.5 Hz, CH), 6.69 (s, 4H, ArH); ^31^P {^1^H} NMR (D_2_O, 243 MHz): δ 15.47 (s); HRMS–ESI: *m/z* [M-4H+3Na]^−^ calcd: 520.9028; found: 520.9044; WG12592A: ^1^H NMR (D_2_O, 600 MHz): δ 4.07 (t, 2H, *J* = 19.9 Hz, CH), 6.81 (d, 2H, *J* = 6.5 Hz, ArH), 7.34–7.38 (m, 4H, ArH); ^31^P {^1^H} NMR (D_2_O, 243 MHz): δ 15.46 (s); HRMS–ESI: m/z [M-4H+3Na]^−^ calcd: 570.9185; found: 570.9193. Copies of ^1^H, ^31^P NMR and HRMS-ESI spectra are available in Supplementary Materials (Figures S1–S6).

### 4.2. Animals

For toxicity studies and osteoporosis experiment, eight-week-old female BALB/c mice were purchased from the Center of Experimental Medicine of the Medical University of Bialystok (Bialystok, Poland) and maintained in specific pathogen-free conditions. For ex vivo analyses, six- to eight-week-old male Foxp3 × Balb/c male mice were obtained from the Hirszfeld Institute of Immunology and Experimental Therapy, Wroclaw. The experiments were performed according to EU Directive 2010/63/EU on the protection of animals used for scientific purposes and were approved by the first Local Committee for Experiments with the Use of Laboratory Animals, Wroclaw, Poland (Permission No.: 4/2015 and 111/2017). Mice were maintained in a temperature-controlled specific pathogen-free facility with access to mouse chow (S8435-S023, Sniff Spezialdiaten, Soest, Germany) and water ad libitum.

### 4.3. Toxicity Studies

The single-dose acute intravenous toxicity was evaluated in Balb/c female mice, weighing 17–22 g. Each group of five mice received, respectively, a single dose of 1.75; 5.5; 17.5; 35; 55.5; 175 mg/kg body weight of WG12399C or 1.75; 3.5; 5.5; 17.5 mg/kg body weight of WG12592A. The general behavior of mice and the signs of toxicity were observed continuously for 30 min after the treatment and then every 30 min for 4 h. Thereafter, mice were further observed and weighted once a day up to 14 days. Behavioral changes, clinical signs of toxicity and deaths were recorded. Animals showing signs of severe pain, permanent signs of severe distress or being in agony, were humanely sacrificed, and the time of death was recorded. The LD_50_ values were estimated from the dose-response curves.

Subacute toxicity of WG12399C and WG12592A was examined based on OECD Test Guideline No. 407 with minor modifications. Each experimental group consisted of five Balb/c female mice, weighing 15–20 g. WG12399C and WG12592A were administered intravenously with the following total doses divided into four weekly injections: WG12399C—18, 35, and 50 mg/kg body weight; WG12592A—1, 2, and 4 mg/kg body weight. The doses for the subacute toxicity test were established taking into account the LD_50_. The animals receiving vehicle (saline) served as control. Mice were observed every day for 28 days, and body weight changes were recorded twice a week. Observations included the condition of skin and fur, eyes, any signs of diarrhea, food and water intake, as well as changes in activity or behavior patterns. After 28 days of observation the animals were sacrificed, the major visceral organs such as the heart, liver, spleen, lungs, and kidneys were removed carefully for gross examination.

### 4.4. Ovariectomy-Induced Osteoporosis

The animals were either sham-operated (*n* = 8) or ovariectomized. Ovariectomy was performed under general anesthesia. Mice received an intraperitoneal injection of buprenorphine at the dose of 0.2 mg/kg (VET-AGRO, Lublin, Poland) and were anaesthetized with a mixture of synthetic air and isoflurane (4% for induction and 2–3% *v/v* for maintenance; 200 mL/min; Aerrane isoflurane, Baxter, Guayama, Puerto Ricoc, IL, USA). To induce the estrogen deficiency, ovariectomy was performed by removing both ovaries through the dorsal approach, and the wounds were closed with soluble surgical sutures. Mice that underwent the same surgical procedure without removing the ovaries (SHAM) served as a control. Twenty-four hours after the surgery, buprenorphine (0.1 mg/kg) was injected. The OVX mice were randomly divided into four groups (*n* = 8, each): Untreated ovariectomized mice (OVX), and mice treated with WG12399C, WG12592A, or zoledronate (ZOL). The animals were kept in gangs of four individuals. Ten days after the surgery, mice started receiving the treatment. Mice were administered intravenously with vehicle (saline), WG12399C 60 mg/kg, WG12592A 6 mg/kg, or zoledronate 120 μg/kg divided into six weekly doses. The doses applied in the study were based on previous toxicological tests and did not cause any clinical signs of toxicity. After 56 days from ovariectomy, mice were anesthetized, and blood samples were obtained for further analyses. The animals were then euthanized by cervical translocation. The femurs and tibias were collected post mortem. For micro-computed tomography (μCT) analysis, bones were immediately frozen and stored in −80 °C, while for histological studies, bones were fixed in 70% ethanol.

### 4.5. Micro-Computed Tomography (μCT)

μCT analysis was performed on excised femurs and tibias. Proximal tibial metaphyses, the distal femoral metaphysis and femoral shafts were scanned with an X-ray μCT system (SkyScan 1172, Bruker, Kontich, Belgium). Each sample was registered at resolution of 6 μm with lamp parameters of 51 kV/194 μA by using an additional 0.5 mm Al filter. 3D structural properties were measured using CTAn software. For each long bone (femur and tibia), measurements were taken for two areas: Spongy bone and compact bone. The method of selecting the region and the representative area of analysis (volume of interest (VOI)) was performed in accordance with the guidelines developed by Bruker for testing on small animals [44]. The following parameters were quantitatively analyzed: Bone mineral density (BMD), bone volume (BV/TV), bone surface (BS/TV), trabecular thickness (Tb.Th), trabecular separation (Tb.Sp), and trabecular number (Tb.N). The BMD value of the examined bones was obtained by comparing them with the BMD value of external density phantoms normalized for murine bones.

### 4.6. Examination of Mechanical Properties of Bones

Mechanical test was conducted on mouse femurs by using the four-point bending test. To eliminate changes in the measured values associate with tissue deformation during the test, the measurement was performed using a protocol dedicated to measuring the mechanical properties of small animals [45]. For this purpose, each bone epiphysis was fixed with Duracryl Plus^®^ adhesive in an aluminum sleeve with a diameter of 6 mm. The distance between the lower supports was 12 mm, and the distance between the point where the force is applied and the lower support was 6 mm. The measurement distance of the embedded femur between the aluminum sleeves was 10 mm. Tests were performed on an MTS 858 MiniBionix machine (Eden Prairie, USA), with displacement speed of 1 mm/min. Because of the shape of the femur, the bone cross-section was approximated with an ellipse to determine the mechanical parameters. For each femur, the values of elastic modulus (E), ultimate strength (σ_ult_), and stiffness (k) were determined. All mechanical parameters determined in the four-point bending test were calculated using classical mechanics formulas.

### 4.7. Histology

Tibias were fixed in 70% ethanol for one week. The samples were then decalcified in formic acid-sodium citrate reagent (45% formic acid and 20% sodium citrate, 1:1) for three days and embedded in paraffin. Next, 3-µm-thick sections of proximal metaphyses were made and stained with a Leukocyte Acid Phosphatase (TRAP) Kit (387A-1KT; Sigma-Aldrich). According to the procedure of Sigma-Aldrich, slides were fixed by immersing in a Fixative Solution (citrate solution, acetone, 37% formaldehyde) for 30 s and rinsed thoroughly in deionized water. The slides were then incubated in the TRAP staining solution (deionized water prewarmed to 37 °C, 45 mL; Diazotized Fast Garnet GBC Solution, 1 mL; Naphthol AS-BI Phosphate Solution, 0.5 mL, Acetate Solution, 2 mL; Tartrate Solution, 1 mL) for 1 h at 37 °C protected from light, rinsed in deionized water, counterstained in hematoxylin solution for 2 min, dehydrated through graded alcohols, cleared in xylene, and mounted.

Microphotographs of all the studied tissues were subjected to a computer-assisted image analysis through a computer coupled to a BX53 optical microscope (Olympus, Tokyo, Japan). The microscopy set had the potential to record images and to analyze them digitally. The measurements were carried out using the CellA software (Olympus Soft Imaging Solution GmbH, Munich, Germany).

### 4.8. Measurement of Serum Levels of Bone Turnover Markers

The expression of selected proteins was detected in mouse plasma. Blood specimens were collected during mice euthanasia to heparinized tubes. Next, the blood samples were immediately centrifuged at 2000× *g* for 15 min at 4 °C, and plasma was transferred into fresh tubes. Calcium level was measured in each sample of plasma using the Cobas c 111 z ISE device (Roche Diagnostics Ltd., Rotkreuz, Switzerland). Tartrate-resistant acid phosphatase 5b (TRACP5b), osteocalcin (OC), and cross linked C-telopeptide of type I collagen (CTX-I) levels were estimated in plasma by CLIA kits (Elabscience, Houston, TX, USA) according to the manufacturer’s instructions.

### 4.9. Cell Culture

Bone marrow was isolated from six- to nine-week-old mice by flushing tibias and femurs with α-MEM medium (Thermo Scientific, Waltham, MA, USA) containing antibiotics. Cells were then centrifuged (1300 rpm, 5 min, 4 °C) and resuspended in α-MEM medium with 10% fetal bovine serum (FBS), 100 U/l penicillin, 100 μg/mL streptomycin, and 75 ng/mL M-CSF (Chiron Corp., Emeryville, CA, USA). Primary bone marrow cells were cultured for 48 h, and the floating cells were discarded; the medium was refreshed, and adherent bone marrow-derived macrophages (BMDMs) were cultured for additional 48 h. The cells were then harvested, and osteoclastogenesis or pit assays were performed.

### 4.10. Macrophage Purity

Macrophage culture was tested for purity by cytofluorescence. Briefly, cells were harvested with Cell Dissociation Solution (Sigma-Aldrich, Darmstadt, Germany), centrifuged, resuspended in PBS (IIET, Wroclaw, Poland) with 2% FBS (Sigma-Aldrich), and transferred to separate tubes at the density of 1 × 10^5^/tube. The cells were stained with fluorophore-conjugated antibody against CD11b and/or F4/80 antigen (BD Biosciences, San Jose, CA, USA), a mouse-specific macrophage marker. The samples were stained on ice for 40 min and then washed with PBS and analyzed. Data analysis was performed by flow cytometry using the Diva Software program for data acquisition (BD Biosciences).

### 4.11. In Vitro Osteoclastogenesis Assay

A total of 2 × 10^4^ differentiated BMDMs were seeded in 24-well plates in α-MEM supplemented with 30 ng/mL M-CSF and 40 ng/mL RANKL (R&D Systems, Minneapolis, USA) and treated with BPs at the concentration of 0.919 μM for WG12399C and 0.421 µM for WG12592A. The concentrations of BPs were based on parallel studies on RAW264.7 macrophages. The IC_50_ values determined for RAW264.7 cells in cytotoxic test (according to the protocol used for J774E macrophages [46]) were 9 µM for WG12399C and 4.5 µM for WG12592A, thus 10 times higher than the doses applied in osteoclastogenesis assay. Untreated cells and cells treated with 0.345 μM zoledronic acid served as controls. Media were replaced every alternate day, and cultures were maintained for eight days. Next, the cells were stained for tartrate-resistant acid phosphatase (TRAP) using an Acid Phosphatase Leukocyte Kit (Sigma-Aldrich) according to the manufacturer’s instructions and counterstained with hematoxylin solution Gill No. 3. Microscopic examination was performed and photographs were captured at 100× magnification using a bright field microscope (Olympus IX81) connected to a camera equipped with Olympus Stream Image Analysis software (Olympus Europe Holding GmBH, Hamburg, Germany). TRAP-positive cells with at least three nuclei were counted as osteoclasts. The number of active osteoclasts in the BP-treated groups was compared to the values in the control wells. Two wells were assessed per treatment in five independent experiments.

### 4.12. Pit Formation Assay

A total of 2 × 10^3^ differentiated BMDMs were seeded in 24-well Corning Osteo Assay Surface Microplates (Corning Inc., Corning, NY, USA) in α-MEM supplemented with 10% FBS and treated with 0.919 μM WG12399C or 0.421 µM WG12592A in the presence of 30 ng/mL M-CSF and 40 ng/mL RANKL. Untreated cells and cells treated with 0.345 μM zoledronic acid served as controls. Media were replaced every alternate day, and cultures were maintained for 10 days. Subsequently, osteostrips were sonicated for 15 min and washed with distilled water. Microscopic examination was performed and microphotographs were captured at 100× magnification with a bright field microscope. The area of resorption pits was measured using Olympus Stream Start 1.6.1 program (Olympus). The area of resorption pits in the BP-treated groups was compared to the values in the control wells. Two wells were assessed per treatment in seven independent experiments.

### 4.13. Statistical Evaluation

Statistical analysis was performed using GraphPad Prism 7.01 (GraphPad Software Inc., San Diego, CA, USA). Shapiro–Wilk’s normality test and Bartlett’s test were used to confirm the assumptions for analysis of variance (ANOVA). Tests used for each data analysis are indicated in figure legends. *p* < 0.05 was considered to be statistically significant.

## 5. Conclusions

In summary, we investigated the efficacy of two aminobisphosphonates WG12399C and WG12592A to prevent ovariectomy-induced osteoporosis in mice. Our μCT analysis of tibiae showed that both WG12399C and WG12592A compounds prevent ovariectomy-induced bone density loss and restore bone parameters such as trabecular separation. These beneficial effects are possibly related to the inhibition of osteoclast differentiation and resorbing activity. The biological effects observed in vivo for WG12399C and WG12592A BPs are not as profound as those observed for zolendronate; however, it is worth noting that zoledronate chosen as the reference compound is the most active bisphosphonate applied in the clinic. The study presented in this paper may set a new direction in the research on the anti-osteoporotic activity of aminomethylidenebisphosphonates.

## Figures and Tables

**Table 1:** Comparison of the values of mechanical parameters obtained in the four-point bending test of the femurs.

Group	Fmax [N]	E [GPa]	σ_ult_ [MPa]	k [N*m^2^]
SHAM	10.974 ± 2.156	19.542 ± 0.725	122.035 ± 19.945	0.0052 ± 0.0007
OVX	10.985 ± 1.736	18.438 ± 1.271	114.300 ± 17.533	0.0055 ± 0.0009
WG12399C	10.425 ± 3.075	17.446 ± 0.953	97.475 ± 17.712	0.0058 ± 0.0009
WG12592A	11.139 ± 2.280	19.409 ± 1.283	113.685 ± 18.499	0.0058 ± 0.0005
Zolendronate	10.959 ± 2.354	19.220 ± 2.475	102.262 ± 19.092	0.0065 ± 0.002

Fmax—maximum bending strength, (E)—elasticity, (σ_ult_)—ultimate strength, (k)—stiffness. Data are shown as mean ± SD.

**Table 2:** Comparison of the mean values of the area moment of inertia obtained in the mid-section of the femoral shaft in the microtomography (I_polar) measurements, and the area moment of inertia measured as the area of the ellipse at the bone fracture site in the four-point bending test (I_ellipse).

Group	I_polar [m^4^]	I_ellipse [m^4^]
SHAM	1.94 × 10^−13^ ± 0.19	2.67 × 10^−13^ ± 0.40
OVX	2.01 × 10^−13^ ± 0.27	2.97 × 10^−13^ ± 0.51
WG12399C	1.95 × 10^−13^ ± 0.20	3.32 × 10^−13^ ± 0.52
WG12592A	2.19 × 10^−13^ ± 0.33	2.99 × 10^−13^ ± 0.29
Zolendronate	2.32 × 10^−13^ ± 0.19	3.38 × 10^−13^ ± 0.67

Data are shown as mean ± SD.

## Data Availability

Data is contained within the article or Supplementary Material.

## Supplementary Materials

The following are available online at https://www.mdpi.com/article/10.3390/ijms22179590/s1, Synthesis of starting bisphosphonic acids, Figure S1. ^31^P{^1^H} NMR spectrum of WG12399C in D_2_O, Figure S2. ^1^H NMR spectrum of WG12399C in D_2_O, Figure S3. ^31^P{^1^H} NMR spectrum of WG12592A in D_2_O, Figure S4. ^1^H NMR spectrum of WG12592A in D_2_O, Figure S5 HRMS-ESI spectrum of WG12399C, Figure S6 HRMS-ESI spectrum of WG12592A, Figure S7 Body weight changes in mice receiving WG12399C and WG12592A, Figure S8. The effect of bisphosphonates on trabecular bone architecture of the distal femoral metaphysis, Figure S9. The effect of bisphosphonates on cortical bone architecture of the femoral shaft.

## References

[1] Feng X., McDonald J.M. (2011). Disorders of Bone Remodeling. Annu. Rev. Pathol. Mech. Dis..

[2] Cummings S.R., Melton L.J. (2002). Osteoporosis I: Epidemiology and outcomes of osteoporotic fractures. Lancet.

[3] Reginster J.Y., Burlet N. (2006). Osteoporosis: A still increasing prevalence. Bone.

[4] Riggs B.L., Khosla S., Melton L.J. (1998). A Unitary Model for Involutional Osteoporosis: Estrogen Deficiency Causes Both Type I and Type II Osteoporosis in Postmenopausal Women and Contributes to Bone Loss in Aging Men. J. Bone Miner. Res..

[5] Pietschmann P., Rauner M., Sipos W., Kerschan-Schindl K. (2009). Osteoporosis: An Age-Related and Gender-Specific Disease—A Mini-Review. Gerontology.

[6] Chen L.R., Ko N.Y., Chen K.H. (2019). Medical treatment for osteoporosis: From molecular to clinical opinions. Int. J. Mol. Sci..

[7] Lawson M.A., Xia Z., Barnett B.L., Triffitt J.T., Phipps R.J., Dunford J.E., Locklin R.M., Ebetino F.H., Russell R.G.G. (2010). Differences between bisphosphonates in binding affinities for hydroxyapatite. J. Biomed. Mater. Res.-Part B Appl. Biomater..

[8] van Beek E., Löwik C., Que I., Papapoulos S. (2009). Dissociation of binding and antiresorptive properties of hydroxybisphosphonates by substitution of the hydroxyl with an amino group. J. Bone Miner. Res..

[9] Van Beek E.R., Löwik C.W.G.M., Ebetino F.H., Papapoulos S.E. (1998). Binding and antiresorptive properties of heterocycle-containing bisphosphonate analogs: Structure-activity relationships. Bone.

[10] Li B., Chau J.F.L., Wang X., Leong W.F. (2011). Bisphosphonates, specific inhibitors of osteoclast function and a class of drugs for osteoporosis therapy. J. Cell. Biochem..

[11] Sundquist K., Lakkakorpi P., Wallmark B., Väänänen K. (1990). Inhibition of osteoclast proton transport by bafilomycin A1 abolishes bone resorption. Biochem. Biophys. Res. Commun..

[12] Luckman S.P., Hughes D.E., Coxon F.P., Russell R.G.G., Rogers M.J., Rogers M.J. (1998). Nitrogen-Containing Bisphosphonates Inhibit the Mevalonate Pathway and Prevent Post-Translational Prenylation of GTP-Binding Proteins, Including Ras. J. Bone Miner. Res..

[13] Goldeman W., Nasulewicz-Goldeman A. (2014). Synthesis and antiproliferative activity of aromatic and aliphatic bis[aminomethylidene(bisphosphonic)] acids. Bioorganic Med. Chem. Lett..

[14] Nasulewicz-Goldeman A., Goldeman W., Mrówczyńska E., Wietrzyk J. (2019). Biological effects of aromatic bis[aminomethylidenebis(phosphonic)] acids in osteoclast precursors in vitro. Chem. Biol. Drug Des..

[15] Banu J. (2011). The Ovariectomized Mice and Rats. Osteoporosis Research.

[16] Holstein J.H., Garcia P., Histing T., Kristen A., Scheuer C., Menger M.D., Pohlemann T. (2009). Advances in the establishment of defined mouse models for the study of fracture healing and bone regeneration. J. Orthop. Trauma.

[17] Weitzmann M.N., Pacifici R. (2006). Estrogen deficiency and bone loss: An inflammatory tale. J. Clin. Investig..

[18] Russell R.G.G., Watts N.B., Ebetino F.H., Rogers M.J. (2008). Mechanisms of action of bisphosphonates: Similarities and differences and their potential influence on clinical efficacy. Osteoporos. Int..

[19] Song L., Bi Y.-N., Zhang P.-Y., Yuan X.-M., Liu Y., Zhang Y., Huang J.-Y., Zhou K. (2017). Optimization of the Time Window of Interest in Ovariectomized Imprinting Control Region Mice for Antiosteoporosis Research. BioMed Res. Int..

[20] Ottewell P.D., Wang N., Meek J., Fowles C.A., Croucher P.I., Eaton C.L., Holen I. (2014). Castration-induced bone loss triggers growth of disseminated prostate cancer cells in bone. Endocr. Relat. Cancer.

[21] Gasser J.A., Ingold P., Venturiere A., Shen V., Green J.R. (2007). Long-Term Protective Effects of Zoledronic Acid on Cancellous and Cortical Bone in the Ovariectomized Rat. J. Bone Miner. Res..

[22] Recker R.R., Delmas P.D., Halse J., Reid I.R., Boonen S., Garcia-Hernandez P.A., Supronik J., Lewiecki E.M., Ochoa L., Miller P. (2008). Effects of intravenous zoledronic acid once yearly on bone remodeling and bone structure. J. Bone Miner. Res..

[23] Bauss F., Lalla S., Endele R., Hothorn L.A. (2002). Effects of treatment with ibandronate on bone mass, architecture, biomechanical properties, and bone concentration of ibandronate in ovariectomized aged rats. J. Rheumatol..

[24] Ottewell P.D., Wang N., Brown H.K., Reeves K.J., Fowles C.A., Croucher P.I., Eaton C.L., Holen I. (2014). Zoledronic acid has differential antitumor activity in the pre- and postmenopausal bone microenvironment in vivo. Clin. Cancer Res..

[25] Kim J., Lee S., Kang S., Moon C., Kim J.-C., Jung U., Jo S.-K., Jang J.-S., Kim S.-H. (2016). Evaluation of the Efficacy of Zoledronic Acid and Amifostine on Radiation-induced Bone Loss in Mice. J. Radiat. Prot. Res..

[26] Parkinson I.H., Fazzalari N.L. (2003). Interrelationships Between Structural Parameters of Cancellous Bone Reveal Accelerated Structural Change at Low Bone Volume. J. Bone Miner. Res..

[27] Jindal M. (2018). Bone Density versus Bone Quality as a Predictor of Bone Strength. Orthop. Rheumatol. Open Access J..

[28] Nikodem A., Ścigała K. (2010). Impact of Some External Factors on the Values of Mechanical Parameters Determined in Tests on Bone Tissue. Acta Bioeng. Biomech..

[29] Roelofs A.J., Stewart C.A., Sun S., Błazewska K.M., Kashemirov B.A., McKenna C.E., Russell R.G.G., Rogers M.J., Lundy M.W., Ebetino F.H. (2012). Influence of bone affinity on the skeletal distribution of fluorescently labeled bisphosphonates in vivo. J. Bone Miner. Res..

[30] Khosla S., Oursler M.J., Monroe D.G. (2012). Estrogen and the skeleton. Trends Endocrinol. Metab..

[31] Nakamura T., Imai Y., Matsumoto T., Sato S., Takeuchi K., Igarashi K., Harada Y., Azuma Y., Krust A., Yamamoto Y. (2007). Estrogen Prevents Bone Loss via Estrogen Receptor α and Induction of Fas Ligand in Osteoclasts. Cell.

[32] Srivastava S., Toraldo G., Weitzmann M.N., Cenci S., Ross F.P., Pacifici R. (2001). Estrogen Decreases Osteoclast Formation by Down-regulating Receptor Activator of NF-κB Ligand (RANKL)-induced JNK Activation. J. Biol. Chem..

[33] Eghbali-Fatourechi G., Khosla S., Sanyal A., Boyle W.J., Lacey D.L., Riggs B.L. (2003). Role of RANK ligand in mediating increased bone resorption in early postmenopausal women. J. Clin. Investig..

[34] Hofbauer L.C., Khosla S., Dunstan C.R., Lacey D.L., Spelsberg T.C., Riggs B.L. (1999). Estrogen Stimulates Gene Expression and Protein Production of Osteoprotegerin in Human Osteoblastic Cells*. Endocrinology.

[35] Zhu S., He H., Gao C., Luo G., Xie Y., Wang H., Tian L., Chen X., Yu X., He C. (2018). Ovariectomy-induced bone loss in TNFα and IL6 gene knockout mice is regulated by different mechanisms. J. Mol. Endocrinol..

[36] Ihn H.J., Kim J.A., Lim S., Nam S.H., Hwang S.H., Lim J., Kim G.Y., Choi Y.H., Jeon Y.J., Lee B.J. (2019). Fermented Oyster Extract prevents Ovariectomy-Induced bone loss and suppresses Osteoclastogenesis. Nutrients.

[37] Zhang Y., Guan H., Li J., Fang Z., Chen W., Li F. (2015). Amlexanox suppresses osteoclastogenesis and prevents ovariectomy-induced bone loss. Sci. Rep..

[38] Wang X., Liang T., Zhu Y., Qiu J., Qiu X., Lian C., Gao B., Peng Y., Liang A., Zhou H. (2019). Melatonin prevents bone destruction in mice with retinoic acid-induced osteoporosis. Mol. Med..

[39] Ito M., Amizuka N., Nakajima T., Ozawa H. (1999). Ultrastructural and cytochemical studies on cell death of osteoclasts induced by bisphosphonate treatment. Bone.

[40] Jobke B., Milovanovic P., Amling M., Busse B. (2014). Bisphosphonate-osteoclasts: Changes in osteoclast morphology and function induced by antiresorptive nitrogen-containing bisphosphonate treatment in osteoporosis patients. Bone.

[41] Nagaoka Y., Kajiya H., Ozeki S., Ikebe T., Okabe K. (2015). Mevalonates restore zoledronic acid-induced osteoclastogenesis inhibition. J. Dent. Res..

[42] Hirayama T., Danks L., Sabokbar A., Athanasou N.A. (2002). Osteoclast formation and activity in the pathogenesis of osteoporosis in rheumatoid arthritis. Rheumatology.

[43] Singh S.K., Manne N., Ray P.C., Pal M. (2008). Synthesis of imidazol-1-yl-acetic acid hydrochloride: A key intermediate for zoledronic acid. Beilstein J. Org. Chem..

[44] Bouxsein M.L., Boyd S.K., Christiansen B.A., Guldberg R.E., Jepsen K.J., Müller R. (2010). Guidelines for assessment of bone microstructure in rodents using micro-computed tomography. J. Bone Miner. Res..

[45] Nowak B., Matuszewska A., Nikodem A., Filipiak J., Landwójtowicz M., Sadanowicz E., Jędrzejuk D., Rzeszutko M., Zduniak K., Piasecki T. (2017). Oral administration of kaempferol inhibits bone loss in rat model of ovariectomy-induced osteopenia. Pharmacol. Rep..

[46] Goldeman W., Nasulewicz-Goldeman A. (2015). Synthesis and biological evaluation of aminomethylidenebisphosphonic derivatives of β-arylethylamines. Tetrahedron.

