# Supplementary material for: Aromatic Bis[aminomethylidenebis(phosphonic)] Acids Prevent Ovariectomy-Induced Bone Loss and Suppress Osteoclastogenesis in Mice
Source: Int J Mol Sci. 2021 Sep 3;22(17):9590. doi: 10.3390/ijms22179590 (PMC8430618; doi:10.3390/ijms22179590)
Supplement: Supplementary file 1 [file ijms-22-09590-s001.zip › ijms-1353779-supplementary.pdf]

Supplementary Materials to the article

Tables of contents

Synthesis of free benzene-1,4-bis[aminomethylidene(bisphosphonic)] acid and naphthalene-1,5-bis-[aminomethylidene(bisphosphonic)]…………………………...2

Fig. S1. $^{31}P\{^{1}H\}$ NMR spectrum of WG12399C in D_2O……………………………………4

Fig. S2. ^{1}H NMR spectrum of WG12399C in D_2O…….…………………..…….........…4

Fig. S3. $^{31}P\{^{1}H\}$ NMR spectrum of WG12592A in D_2O……………………………..…...5

Fig. S4. ^{1}H NMR spectrum of WG12592A in D_2O…..……………...…………………..5

Fig. S5. HRMS-ESI spectrum of WG12399C…..…………………...…………………....6

Fig. S6. HRMS-ESI spectrum of WG12592A…..…………...……………………...…...7

Fig. S7. Body weight changes in mice receiving WG12399C and WG12592A…………8

Fig. S8. The effect of bisphosphonates on trabecular bone architecture of the distal femoral metaphysis…….……………………………………………………………….….9

Fig. S9. The effect of bisphosphonates on cortical bone architecture of the femoral shaft……………………………………………………………………………..…11

Supplementary references……………………………………………………………12

Synthesis of free benzene-1,4-bis[aminomethylidene(bisphosphonic)] acid and naphthalene-1,5-bis-[aminomethylidene(bisphosphonic)]

I step: synthesis of octaethyl benzene-1,4-bis[aminomethylidene(bisphosphonate)] **1a** and octaethyl naphthyl-1,5-bis[aminomethylidene(bisphosphonate)] **1b**.

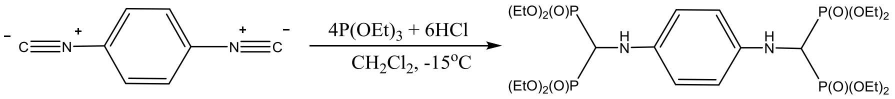

1a

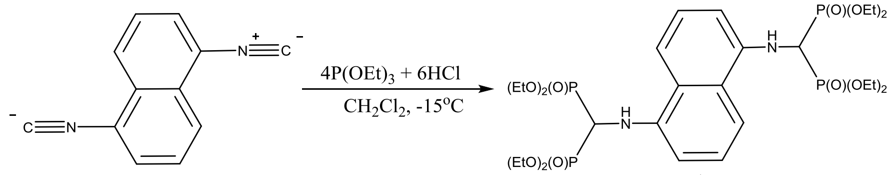

1b

To a cold (*ca.* -15°C, NaCl-ice bath) solution of 1,4-diisocyanobenzene (0.048 mol, 6.2 g) or 1,5-diisocyanonaphthalene (0.048 mol, 8.5 g) and triethyl phosphite (33.2 g, 0.20 mol, Sigma-Aldrich) in dry dichloromethane (300 ml) a cold solution of *ca.* 4 M HCl in 1,4-dioxane (0.3 mol, 75 ml) was added during about 30 min and the mixture was stirred for additional 1 h at the same temperature. The reaction mixture was washed with saturated $NaHCO_3$ solution (8×50 ml), dried over anhydrous Na_2SO_4 and concentrated *in vacuo*. The residue was treated with diethyl ether/petroleum ether (150 ml, 1:2, v/v) in the case of **1a** and diethyl ether (200 ml) in the case of **1b**. The resulting solids were filtered, washed intensively with diethyl ether/petroleum ether (4×30 ml, 1:2, v/v) or diethyl ether (5×20 ml), respectively and dried in air giving octaethyl benzene-1,4-bis[aminomethylidene(bisphosphonate)] (15.9 g, 49%) **1a** and octaethyl naphthyl-1,5-bis[aminomethylidene(bisphosphonate)] **1b** (26.7 g, 76%). Both octaethyl esters were used in next step without further purification. The structure of **1a** and **1b** was confirmed by ^{31}P and ^{1}H NMR spectroscopy. **1a**: ^{31}P NMR {^{1}H} ($CDCl_3$, 121 MHz): δ 18.0 (s); ^{1}H NMR ($CDCl_3$, 300 MHz): δ 1.27 (t, *J*=7.1Hz,12H, OCH_2CH_3), 1.30 (t, *J*=7.1Hz, 12H, OCH_2CH_3), 3.80 (bs, 2H, NH), 4.07 (bt, *J*=22.2Hz, 2H, CHP) 4.14-4.22 (m, 16H, OCH_2), 6.63 (s, 4H, ArH); **2b**: ^{31}P NMR {^{1}H} ($CDCl_3$, 121 MHz): δ 17.5 (s); ^{1}H NMR ($CDCl_3$, 300 MHz): δ 1.22 (t, *J*=7.0Hz, 12H, OCH_2CH_3), 1.29 (t, *J*=7.0Hz, 12H, OCH_2CH_3), 4.08-4.17 (m, 4H, OCH_2), 4.18-4.29 (m, 12H, OCH_2), 4.42 (dt, 2H, J=10.2Hz, J=21.6Hz, CHP), 4.91 (bdt, 2H, J=4.8Hz, J=9.6Hz, NH), 6.80 (t, 2H, J=4.0Hz, ArH), 7.36 (m, 4H, ArH). NMR data are in agreement with those reported in our previous paper [1].

II step: synthesis of benzene-1,4-bis[aminomethylidene(bisphosphonic)] acid **2a** and naphthalene-1,5-bis-[aminomethylidene(bisphosphonic)] acid **2b**.

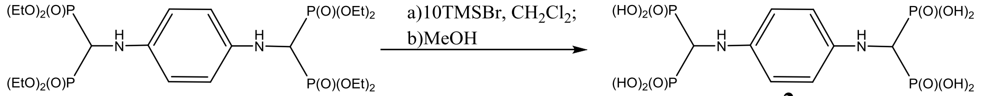

2a

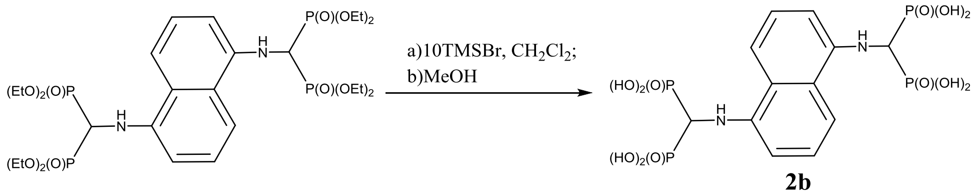

2b

To a cold (*ca.* 0°C, ice bath) solution of octaethyl benzene-1,4-bis[aminomethylidene(bisphosphonate)] **1a** (0.020 mol, 13.6 g) or octaethyl naphthyl-1,5-bis[aminomethylidene(bisphosphonate)] **1b** (0.020 mol, 14.6 g) in dry dichloromethane (200 ml) a bromotrimethylsilane (26.4 ml, 0.20 mol) was added dropwise over 30 min. The mixture was stirred for about 1 h at 0°C and overnight at room temperature. Then was concentrated *in vacuo*, the resulting residue was treated with methanol (200 ml) and stirred for about 30 minutes at room temperature. The reaction mixture was evaporated again and a methanol-acetone mixture (300 ml, 2:1, v/v) was added. The resulting solid products were filtered off, washed with a methanol-acetone mixture (3×30 ml, 2:1, v/v), water-acetone mixture (2×50 ml, 1:1, v/v), acetone (2×50 ml) and dried in air giving benzene-1,4-bis[aminomethylidene(bisphosphonic)] acid **2a** (8.2 g, 90%) and naphthalene-1,5-bis-[aminomethylidene(bisphosphonic)] acid **2b** (6.8 g, 67%). Obtained free acids were directly used for the preparation of their tetrasodium salts (WG 12399C and WG12592A).

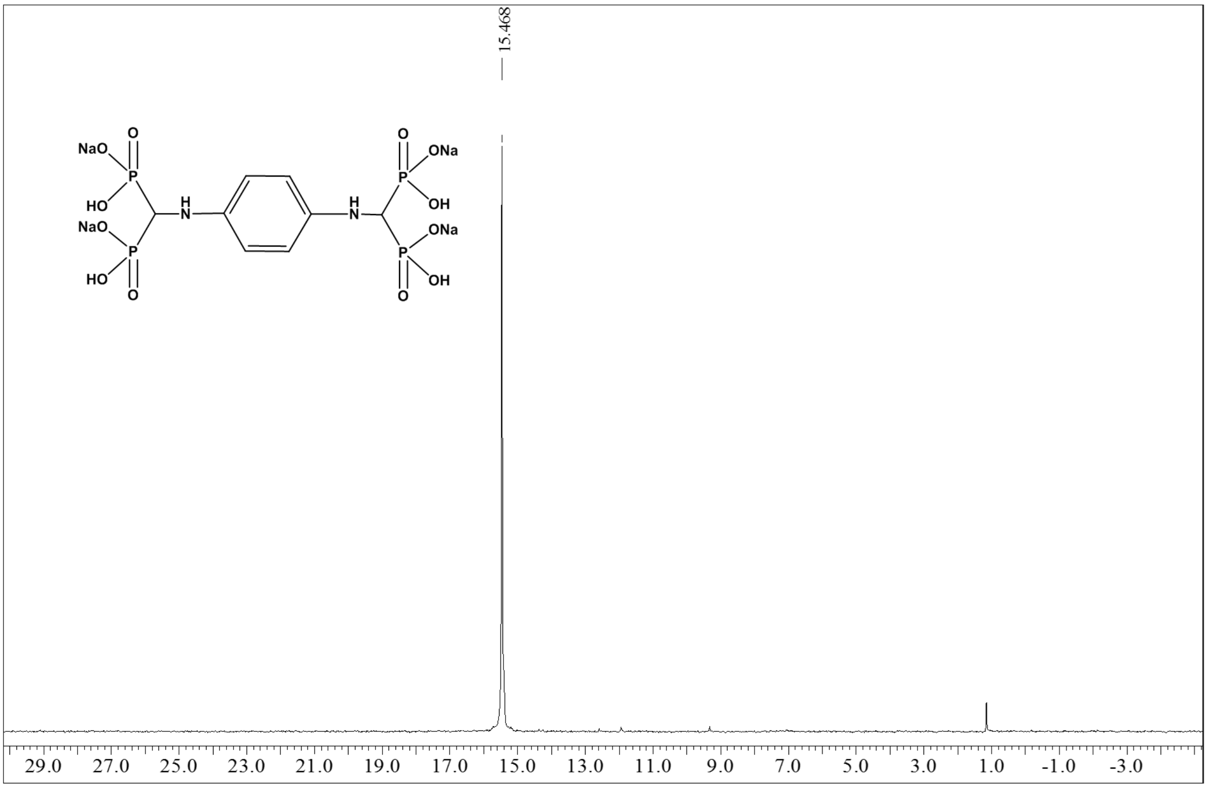

Figure S1. $^{31}P\{^{1}H\}$ NMR spectrum of WG12399C in D_2O.

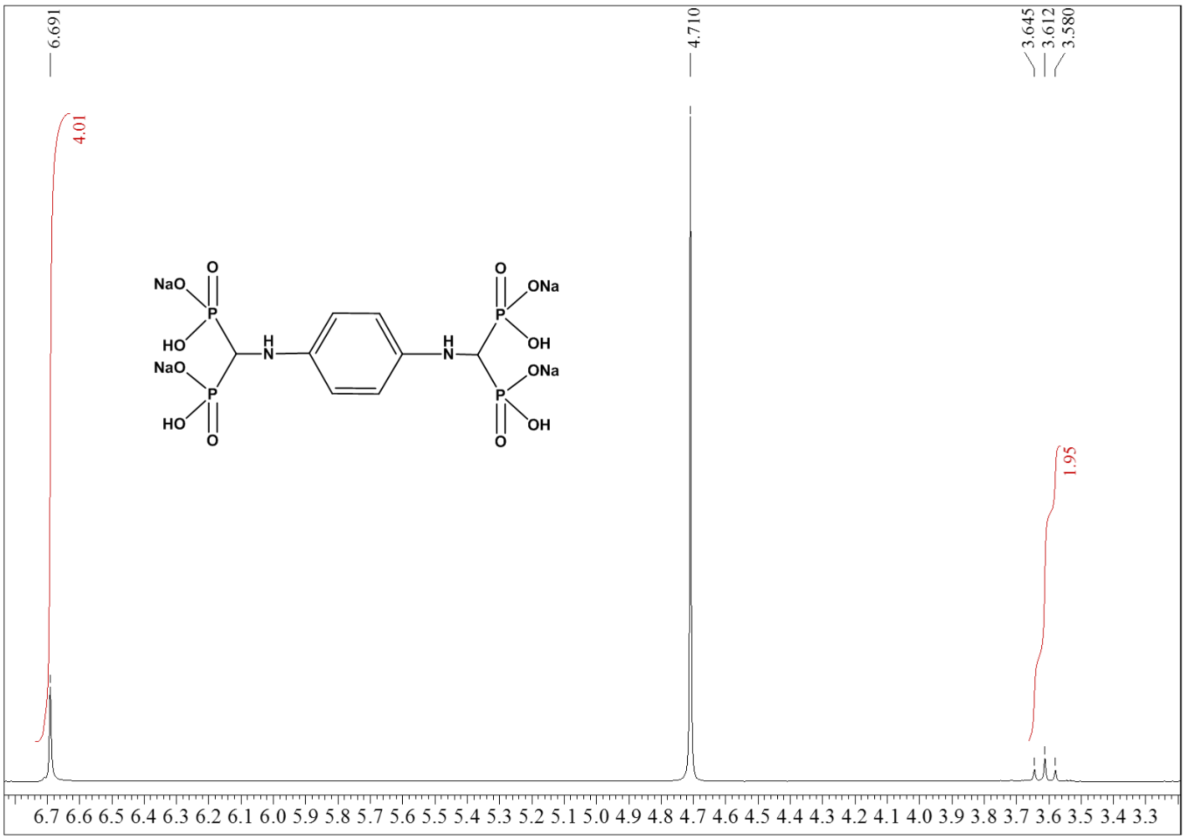

Figure S2. ^{1}H NMR spectrum of WG12399C in D_2O.

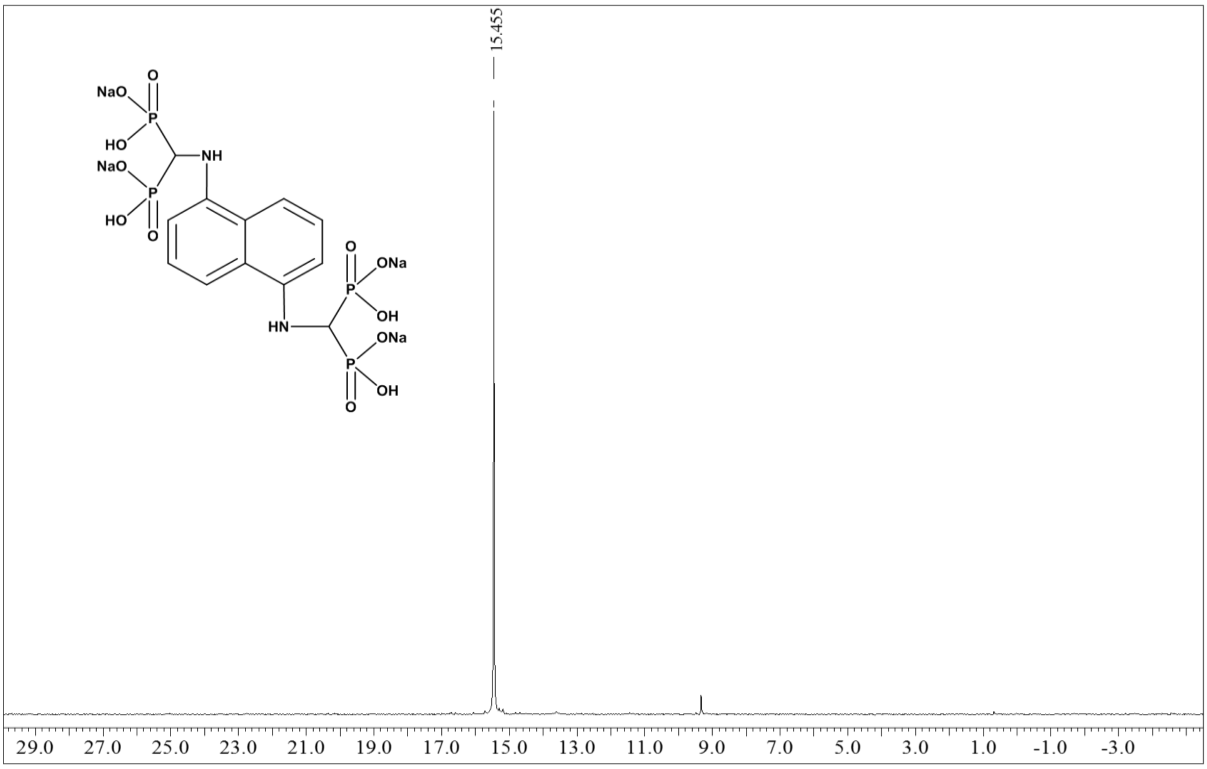

Figure S3. $^{31}P\{^{1}H\}$ NMR spectrum of WG12592A in D_2O.

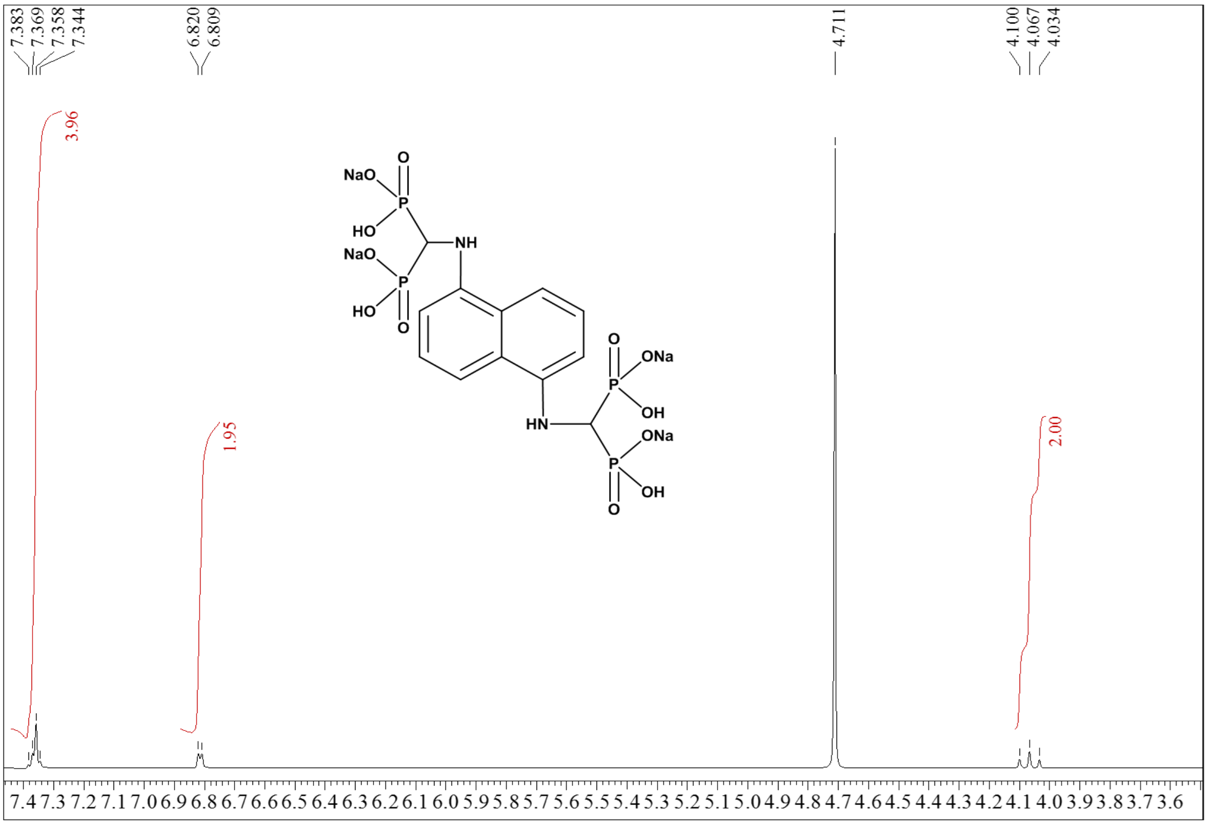

Figure S4. ^{1}H NMR spectrum of WG12592A in D_2O.

A.

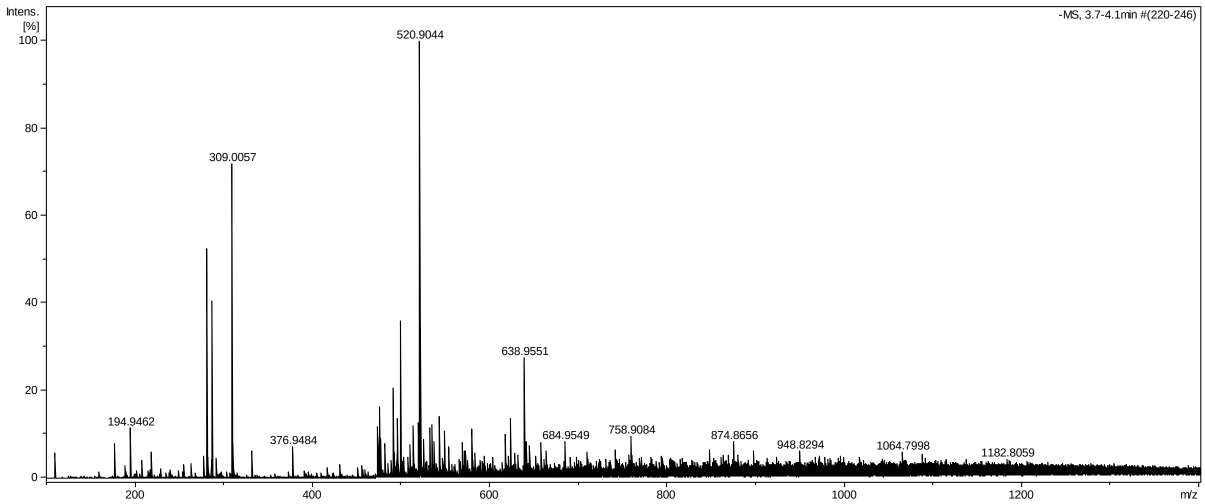

B.

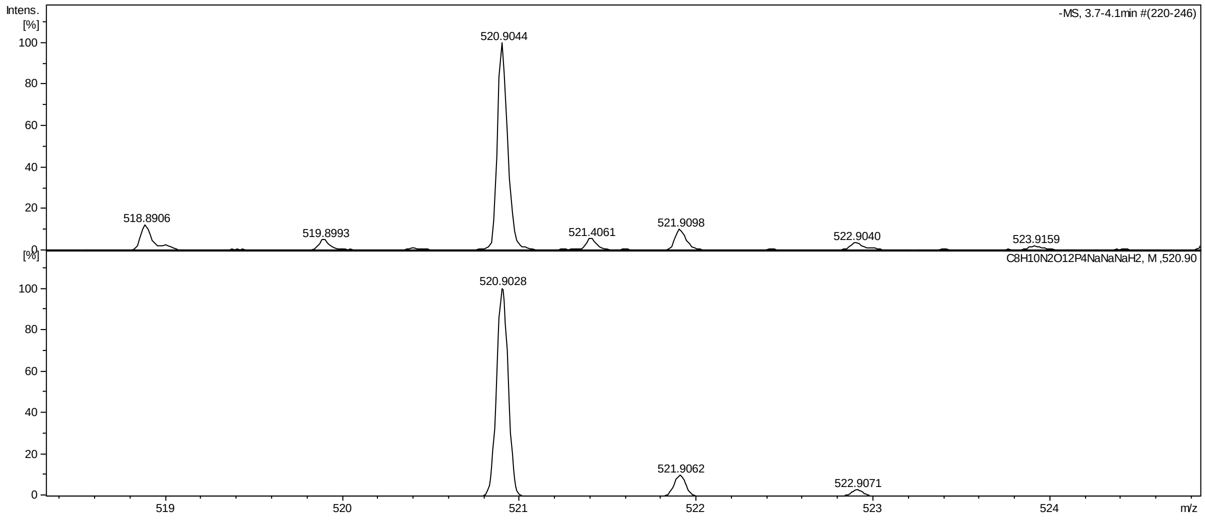

Figure S5. HRMS-ESI spectrum of WG12399C. A. Full HRMS-ESI spectrum. B. Expanded HRMS-ESI spectrum with measured (top) and simulated (bottom) [M-4H+3Na]$^-$ peak.

A.

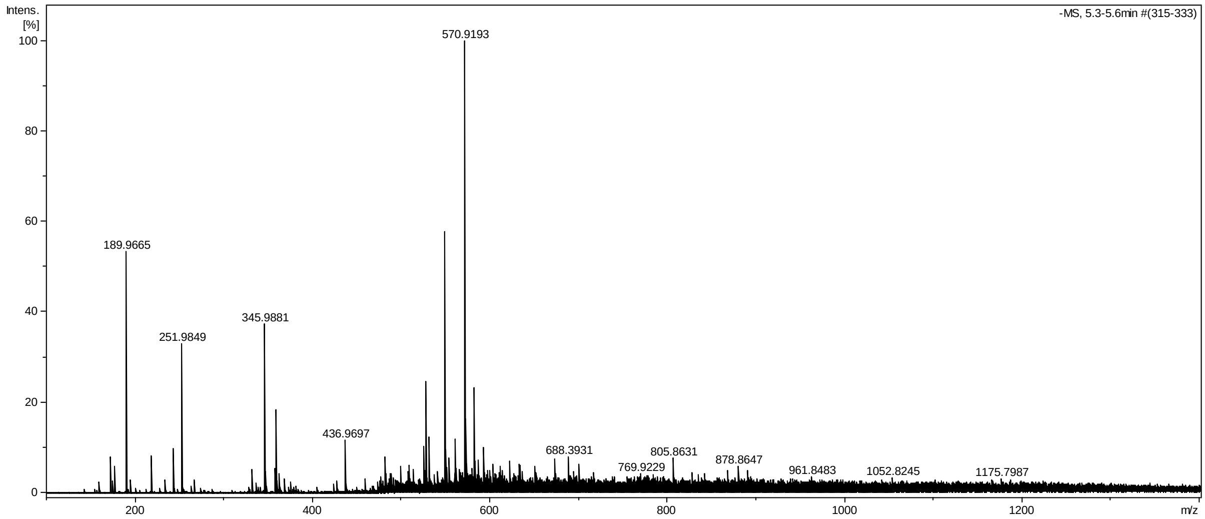

B.

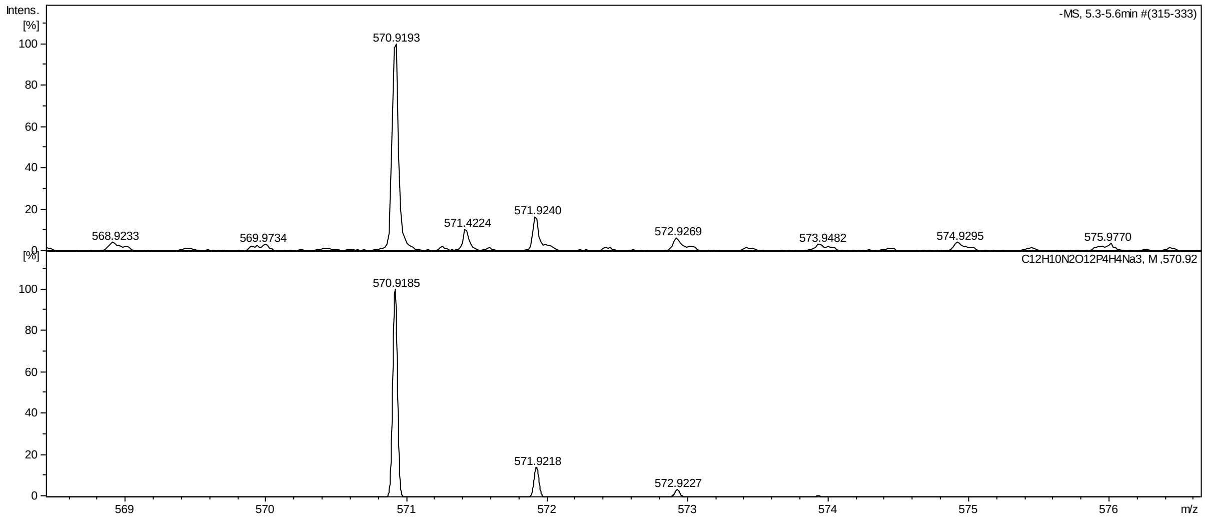

Figure S6. HRMS-ESI spectrum of WG12592A. A. Full HRMS-ESI spectrum. B. Expanded HRMS-ESI spectrum with measured (top) and simulated (bottom) $[M-4H+3Na]^{-}$ peak.

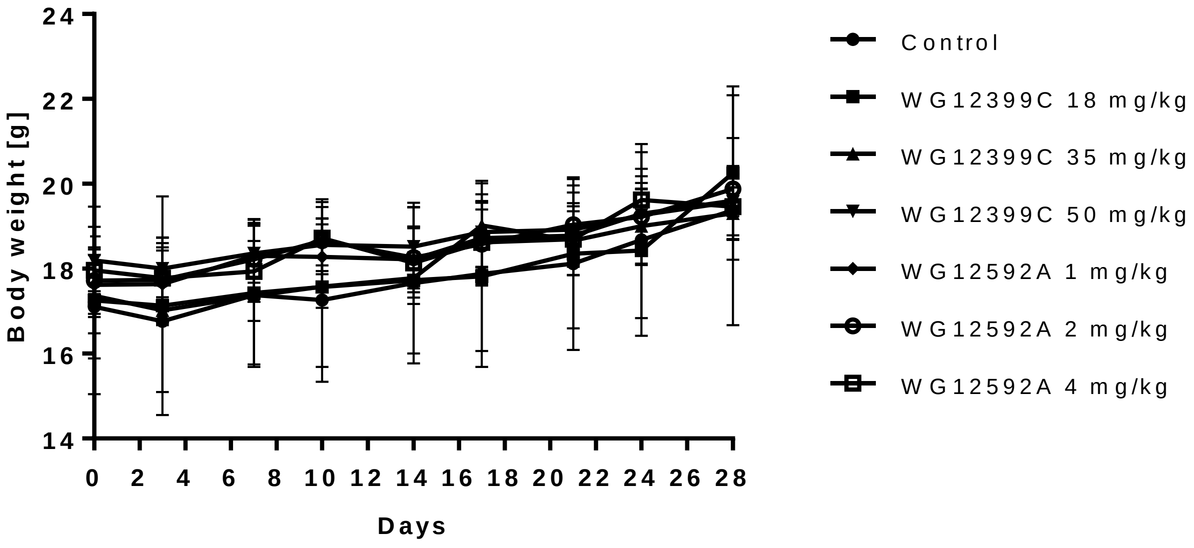

Figure S7. Body weight changes in mice receiving WG12399C and WG12592A. Starting from the day 0 mice were administered intravenously with the following total doses divided into four weekly injections: WG12399C - 18, 35 and 50 mg/kg; WG12592A - 1, 2 and 4 mg/kg. Data are expressed as mean ± standard deviation, n=5.

A.

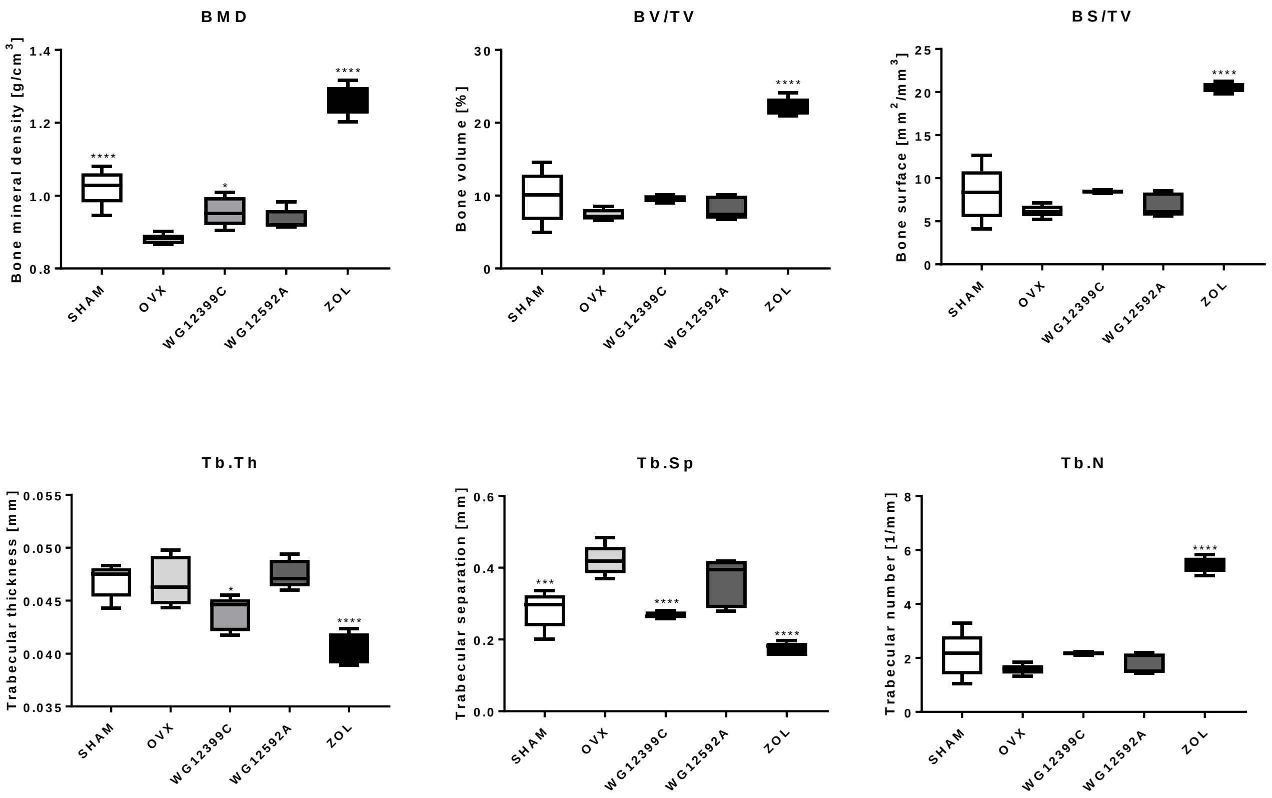
BMD
Bone mineral density [g/cm3]
BV/TV
Bone volume [%]
BS/TV
Bone surface [mm2/mm3]
Tb.Th
Trabecular thickness [mm]
Tb.Sp
Trabecular separation [mm]
Tb.N
Trabecular number [1/mm]
SHAM
OVX
WG12399C
WG12592A
ZOL

B.

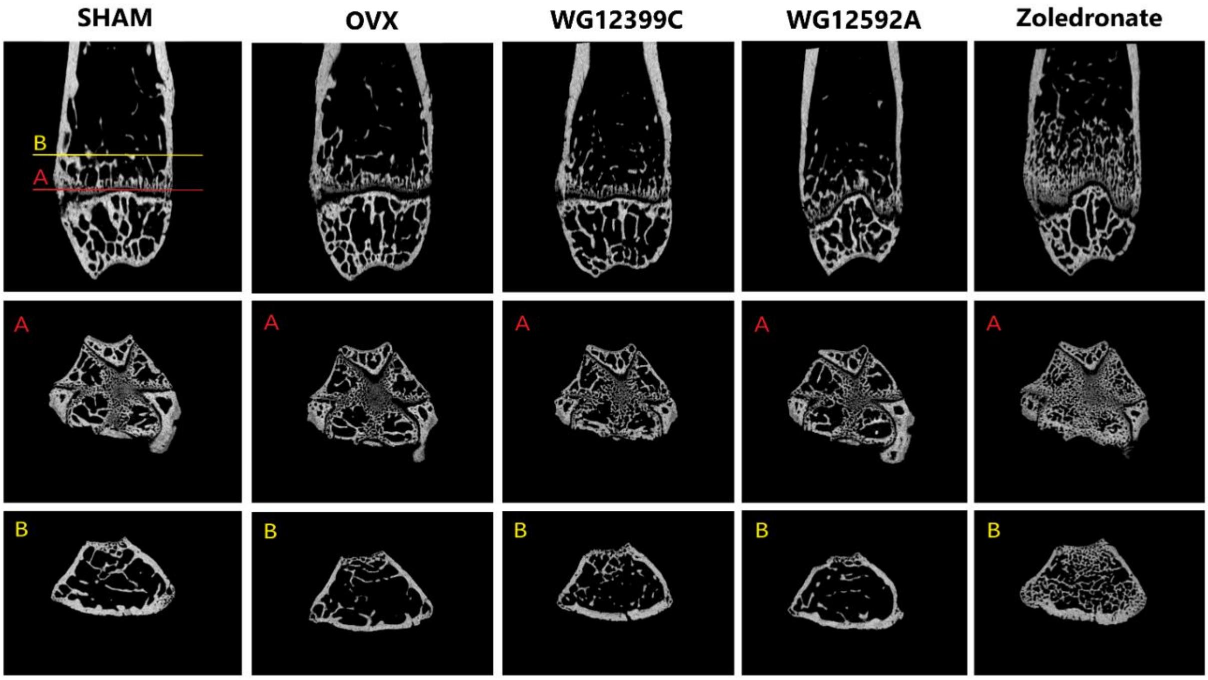
SHAM
OVX
WG12399C
WG12592A
Zoledronate
A
B

Figure S8. The effect of bisphosphonates on trabecular bone architecture of the distal femoral metaphysis. Mice were bilaterally ovariectomized and then administered intravenously with WG12399C 60 mg/kg, WG12592A 6 mg/kg or zoledronate 120 μg/kg in weekly doses. Vehicle-treated (OVX) and sham-operated (SHAM) mice served as controls. A. Histograms representing the structural parameters of the trabecular bone measured with μ-CT: BMD – bone mineral density; BV/TV - bone volume, BS/TV - bone surface, Tb. Th – trabecular thickness, Tb.Sp – trabecular separation, Tb.N – trabecular number. Data are expressed as mean ± standard deviation, n=5, *p<0.05, **p<0.01, ***p<0.001, and ****p<0.0001 vs OVX group were assessed with one-way analysis of variance. B. μ-CT images of the distal femurs (two upper lines, axial view of the metaphyseal region; bottom, longitudinal view).

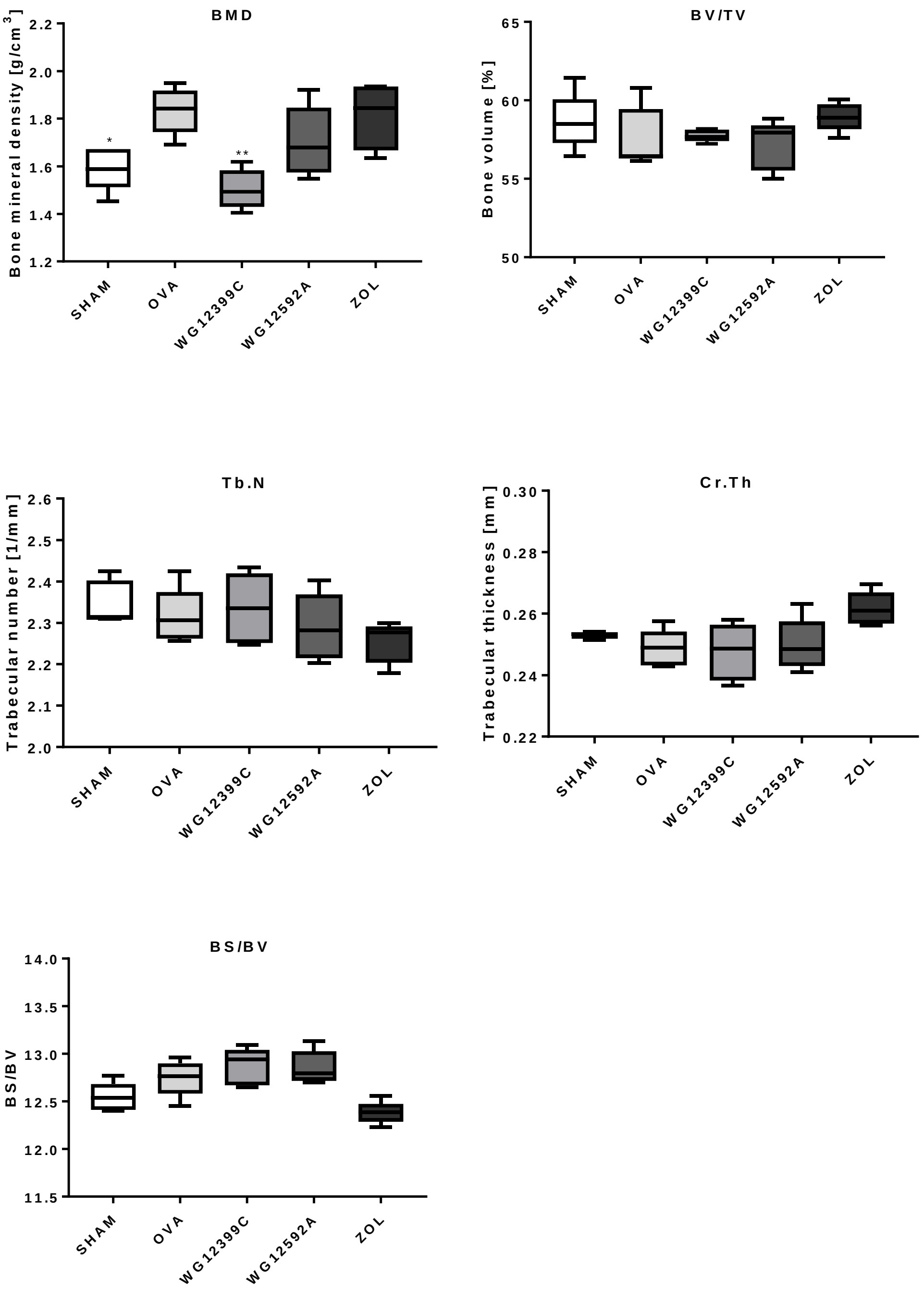

Figure S9. The effect of bisphosphonates on cortical bone architecture of the femoral shaft. Mice were bilaterally ovariectomized and then administered intravenously with WG12399C 60 mg/kg, WG12592A 6 mg/kg or zoledronate 120 μg/kg in weekly doses. Vehicle-treated (OVX) and sham-operated (SHAM) mice served as controls. A. Histograms representing the structural parameters of the cortical bone measured with μ-CT: BMD – bone mineral density; BV/TV - bone volume; Tb.N – trabecular number; Cr.Th – trabecular thickness; BS/BV - bone surface/bone volume ratio. Data are expressed as mean ± standard deviation, n=5, **p<0.01 vs OVX group were assessed with one-way analysis of variance.

Supplementary references:

1. Goldeman, W.; Nasulewicz-Goldeman, A. Synthesis and antiproliferative activity of aromatic and aliphatic bis[aminomethylidene(bisphosphonic)] acids. *Bioorganic Med. Chem. Lett.* **2014**, *24*, 3475–3479, doi:10.1016/j.bmcl.2014.05.071.
